# Comparative transcriptomic profiling of human conjunctival epithelial cells and macrophages in response to *Chlamydia trachomatis* genovars A and B in early- and mid-infection cycles

**DOI:** 10.3389/fcimb.2026.1755017

**Published:** 2026-04-22

**Authors:** Ehsan Ghasemian, Martin J. Holland

**Affiliations:** 1 Institute of Microbiology, University Hospital Center and University of Lausanne, Lausanne, Switzerland; 2 Department of Clinical Research, London School of Hygiene & Tropical Medicine, London, United Kingdom

**Keywords:** *Chlamydia trachomatis*, human conjunctival epithelial cells, macrophages, trachoma, transcriptomics

## Abstract

*Chlamydia trachomatis* (Ct), an obligate intracellular bacterium, is the primary infectious cause of blindness through trachoma. Ct undergoes a unique biphasic developmental cycle between infectious elementary bodies and replicating reticulate bodies, manipulating host cells via secreted effector proteins. Whilst previous studies have characterised host-pathogen interactions including transcriptomes of urogenital Ct genovars E and L2, limited studies exist on ocular Ct genovars. This study examined transcriptomic responses of human conjunctival epithelial (HCjE) cells and PMA-differentiated THP-1 macrophages to infection with ocular Ct strains A/2497 or B/Tunis864 (live or heat-inactivated) at 4 and 24 hours post-infection (hpi). Transcriptomic profiling was performed using RNA sequencing, with differential gene expression analysis. Pathway enrichment analyses were performed to identify strain-specific and cell type-specific transcriptional signatures. HCjE cells exhibited progressive transcriptional activation, with differentially expressed genes (DEGs) increasing from 4 to 24 hpi (144 to 259), whilst THP-1 macrophages showed temporal attenuation (391 to 154). B/Tunis864 consistently elicited stronger responses than A/2497 in HCjE cells at both time points (152 *vs*. 54 DEGs at 4 hpi; 259 *vs*. 83 at 24 hpi). Conversely, THP-1 macrophages showed stronger responses to A/2497 than B/Tunis864 at both time points (599 *vs*. 376 DEGs at 4 hpi; 166 *vs*. 114 at 24 hpi). HCjE cells demonstrated markedly higher proportions of strain-specific DEGs and pathways compared to macrophages. B/Tunis864 infection in HCjE cells induced pronounced interferon (IFN)-stimulated gene signatures, particularly at 24 hpi. This study revealed contrasting temporal and strain-specific patterns: THP-1 macrophages showed peak-then-decline, strain-invariant responses, whilst HCjE cells exhibited progressive activation with predominantly strain-specific programmes. Enhanced IFN-stimulated gene and inflammatory pathway activation by B/Tunis864 in HCjE cells may provide molecular insights into genovar B-associated trachoma severity.

## Introduction

*Chlamydia trachomatis* (Ct) is an obligate intracellular bacterial pathogen that primarily infects ocular and urogenital mucosal epithelia (Brunham and Rey-Ladino, 2005; Solomon et al., 2022a). Ct is the etiological agent of trachoma, which represents the leading infectious cause of preventable blindness worldwide. It is also responsible for the most prevalent bacterial sexually transmitted infection (Taylor et al., 2014; Ghasemian et al., 2023). The pathological sequelae of chlamydial infection result from dysregulated host inflammatory and immune responses that drive chronic inflammation, progressive tissue remodelling, and ultimately fibrotic scarring of the upper reproductive tract and conjunctival tissues (Brunham and Rey-Ladino, 2005; Hu et al., 2013; Solomon et al., 2022b). These pathological processes are mediated through host-pathogen interactions involving multiple mechanisms. Cell-autonomous immune responses, such as interleukin (IL)-1R-dependent pathways, play a dual role: whilst promoting bacterial clearance, they simultaneously inflict collateral tissue damage that contributes to pathology (Finethy and Coers, 2016). Additionally, Ct infection may induce epithelial-mesenchymal transition (EMT) and stimulate production of extracellular matrix components and collagen I in host epithelial cells, establishing a pro-fibrotic programme that operates concurrently with inflammatory responses (Rajić et al., 2017; Igietseme et al., 2018; Caven and Carabeo, 2023). This dual activation of pro-inflammatory and pro-fibrotic signalling pathways in infected epithelial cells represents an expanded cellular paradigm of chlamydial pathogenesis, wherein tissue scarring results not only from collateral inflammatory damage but also from direct pathogen-induced fibrotic remodelling (Igietseme et al., 2018). Furthermore, Ct actively manipulates host immune signalling through secreted bacterial effector proteins, exemplified by the deubiquitinating protease ChlaDub1, which inhibits nuclear factor kappa B (NF-κB) activation, a regulator of inflammatory responses and cytokine production, thereby facilitating bacterial survival and persistence (Le Negrate et al., 2008; Rodrigues et al., 2025).

Ct has a unique biphasic developmental cycle, alternating between two morphologically and functionally distinct forms: metabolically inactive, infectious elementary bodies (EBs) and metabolically active, replicating reticulate bodies (RBs) (Yang et al., 2024). The chlamydial developmental cycle can be temporally divided into early (1–8 hours post-infection (hpi)), mid (8–48 hpi), and late (48–72 hpi) phases based on differential gene expression patterns, replicative activity, and host-pathogen interactions (Nicholson et al., 2003; AbdelRahman and Belland, 2005; Elwell et al., 2016; Jury et al., 2023). Infection is initiated when EBs attach to and invade susceptible host cells through receptor-mediated endocytosis (AbdelRahman and Belland, 2005; Richards et al., 2013). Following internalisation, EBs establish residence within membrane-bound inclusion vacuoles that actively evade phagolysosomal fusion, creating a protective intracellular niche where they differentiate into metabolically active RBs. After multiple rounds of binary fission, RBs asynchronously re-differentiate back into infectious EBs, which are subsequently released through either inclusion extrusion or host cell lysis to reinitiate the infection cycle (Clifton et al., 2004; Richards et al., 2013). Throughout this developmental process, Ct employs its type III secretion system (T3SS) to translocate effector proteins directly from the bacterial cytoplasm into the host cell cytosol (Clifton et al., 2004; Subtil et al., 2005; Nans et al., 2015), and secrete virulence factors that manipulate multiple aspects of host cell biology: TarP and TmeA coordinate actin cytoskeleton reorganisation to facilitate invasion (Romero and Carabeo, 2022); IncD and IncV establish Endoplasmic Reticulum-inclusion membrane contact sites essential for intracellular survival and replication (Derré et al., 2011; Murray et al., 2017); whilst GarD, CpoS, and chlamydial protease-like activity factor (CPAF) collectively subvert both innate and adaptive immune responses to ensure bacterial persistence (Tang et al., 2015; Yang et al., 2016b; Yang et al., 2016a; Sixt et al., 2017; Walsh et al., 2022; Meier et al., 2023).

Previous studies by Hayward et al. (Hayward et al., 2019; Hayward et al., 2021) provided insights into host-pathogen interactions during Ct genovar E (CtE) infection through the application of single-cell RNA sequencing and dual RNA-seq approaches to simultaneously analyse host and bacterial transcriptomes. These studies demonstrated that adherence and uptake of Ct rapidly triggers transcriptional reprogramming in infected host cells (Hayward et al., 2019; Hayward et al., 2021). A study by Humphrys et al. (2013) revealed the role of Ct in subverting host immune responses through multiple mechanisms, including downregulation of antimicrobial peptides and mucin expression, attenuation of innate immune signalling and modulation of pathways controlling immune cell recruitment and activation (Humphrys et al., 2013). It was hypothesised that these transcriptional alterations may contribute to progressive tissue damage and pathological sequelae (Humphrys et al., 2013). Furthermore, *in vitro* studies utilising genital CtE and genovar L2 have elucidated the temporal dynamics of host transcriptional modulation, revealing differential regulation of genes involved in metallothionein function, cell cycle control, innate immunity, cytoskeletal organisation, lipid biosynthesis, and cellular stress responses compared to mock-infected controls (Hayward et al., 2019; Dzakah et al., 2021; Caven et al., 2023).

**Figure 1:**
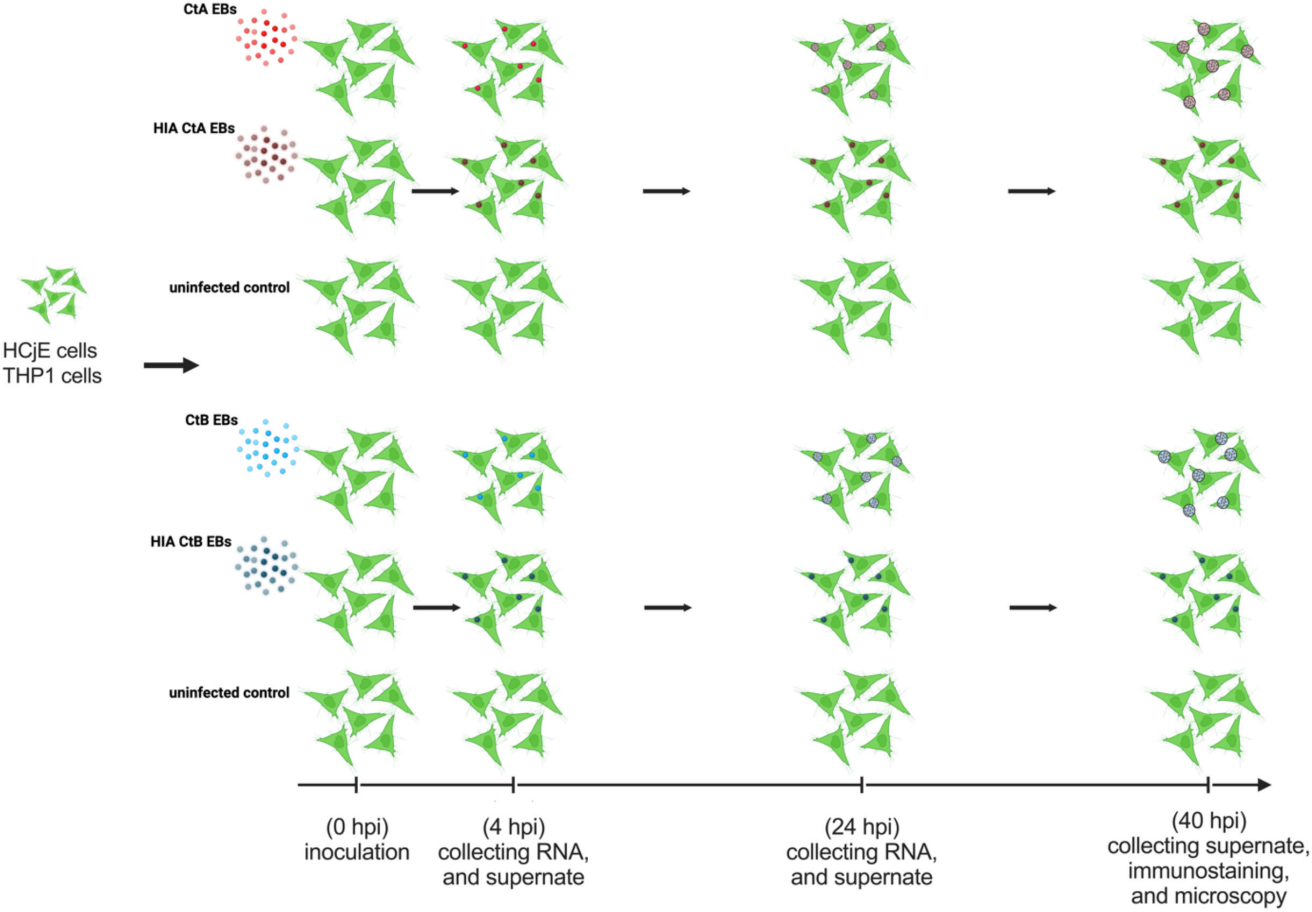
Experimental design. HCjE and THP-1 cells were inoculated with live or HIA CtA or CtB, with uninfected cells serving as negative controls. RNA was extracted at 4 and 24 hpi, and chlamydial infection was assessed by microscopy at 40 hpi. Colour coding: live CtA (red), HIA CtA (dark red), live CtB (blue), HIA CtB (dark blue).

Ct genovars A (CtA), B (CtB) and C are the causative agents of blinding trachoma (Taylor et al., 2014; Harte et al., 2024), and may exhibit different molecular strategies for epithelial cell interaction and immune evasion (Stothard et al., 2003; Carlson et al., 2005; McClarty et al., 2007; Sigalova et al., 2019; Yang et al., 2024). Previous investigations have documented distinct bacterial load kinetics, disease severity, infection duration, and genomic evolutionary pressures between CtA and CtB (Bobo et al., 1997; Miyairi et al., 2006; Andreasen et al., 2008; Kari et al., 2008; Last et al., 2017; Chin et al., 2018; Last et al., 2018; Ghasemian et al., 2021; Ghasemian et al., 2024; Ghasemian et al., 2025). Despite advances in understanding Ct-host interactions, our knowledge of how these ocular genovars modulate host cell transcriptomes remains limited. In this study, we conducted transcriptomic analysis of phorbol 12-myristate 13-acetate (PMA)-activated THP-1 cells and immortalised human conjunctival epithelial (HCjE) cells infected with either live or heat-inactivated (HIA) Ct strain A/2497 or B/Tunis864 at 4 and 24 hpi (**Figure 1**). HCjE cells, are immortalised conjunctival epithelial cells and represent the primary target tissue for ocular Ct infection (Derrick et al., 2015; Dzakah et al., 2021; Ahmad et al., 2025), whilst PMA-activated THP-1 cells model the monocyte-derived macrophages that are recruited to the site of infection (Morrison and Morrison, 2000; Prantner et al., 2009; Lausen et al., 2019). It is known that the initial engagement between macrophages and Ct may determine the overall outcome of the infection since if intracellular elimination in macrophages fails, macrophages may be used as Trojan horses for dissemination of Ct (de Vries et al., 2009; Lausen et al., 2019). Through this study we aim to: (*i*) evaluate the responsiveness of HCjE and THP-1 cells to Ct strains A/2497 and B/Tunis864 infection, representing CtA and CtB, respectively, (*ii*) identify differential host cellular pathway enrichment patterns in response to A/2497 *vs*. B/Tunis864 infection, (*iii*) determine transcriptional specifications associated with early- and mid- developmental stages of Ct, and (*iv*) suggesting genes and pathways underlying the pathogenesis and varied clinical outcomes of trachoma in relation to Ct genovar.

## Materials and methods

### Bacterial stock preparation

HeLa cells monolayers, kindly provided by Professor David Allen (London School of Hygiene and Tropical Medicine), were infected with Ct strains A/2497 (Harris et al., 2012) and B/Tunis864 (Ghasemian and Holland, 2024). For bacterial stock preparation, EBs were propagated in HeLa cell monolayers and subsequently purified using Gastrografin density gradient centrifugation (Faris and Weber, 2019). Purified Ct EB stocks were titrated by serial dilution and reinfection of HeLa cell monolayers, with quantification performed using Pathfinder anti-chlamydial lipopolysaccharide (LPS) immunofluorescent staining (Bio-Rad). Fluorescent imaging and counting of inclusion-forming units (IFUs) were conducted using a Nikon ECLIPSE Ti microscope (Nikon Instruments Inc.). The purified Ct EBs were resuspended in sucrose phosphate glutamate (SPG) buffer and stored at -70 °C until use.

### Inoculation of HCjE and THP-1 cells

HCjE cells, kindly provided by Professor Ilene K. Gipson (Harvard Medical School), were cultured as monolayers in 6-well plates until reaching 90% confluency using Keratinocyte Serum-Free Medium (SFM) (Gibco) supplemented with 25 ug/mL Bovine Pituitary Extract (BPE), 0.2 ng/mL epidermal growth factor (EGF), 0.4 mM CaCl_2_ (Merck), 25 ug/mL gentamicin (Gibco), 20 ug/mL vancomycin (Fisher scientific), and 50 ug/mL amphotericin B (Gibco). THP-1 cells (a human monocyte cell line), kindly provided by a colleague at the London School of Hygiene and Tropical Medicine, were maintained in RPMI 1640 medium (Gibco) supplemented with 10% fetal bovine serum (FBS) (Gibco), 2 mM L-Glutamine (Gibco), 0.05 mM 2-mercaptoethanol (Merck), 25 ug/mL gentamicin (Gibco), 20 ug/mL vancomycin (Fisher scientific), and 50 ug/mL amphotericin B (Gibco). For macrophage differentiation, THP-1 cells were seeded in 6-well plates at 1x 10^6^ cells per well and treated with 0.16 μg/mL PMA (Merck) for 72 h to induce differentiation into macrophage-like cells.

Inoculation medium was prepared specifically for each cell type. For HCjE cells, Dulbecco’s Modified Eagle Medium (DMEM)/F-12 (Gibco) was supplemented with 1% gentamicin (Gibco) and 1% L-glutamine (Gibco). For THP-1 cells, RPMI-1640 (Gibco) medium was supplemented with 10% FBS (Gibco), 2 mM L-glutamine (Gibco), and 1% gentamicin (Gibco). Prior to infection, cell monolayers were washed three times with Hank’s Balanced Salt Solution (HBSS) (Merck). Each cell line was inoculated in biological triplicate with: (*i*) live Ct at MOI 5, (*ii*) HIA Ct at MOI 5, or (*iii*) inoculation medium alone as a mock-infected control. Infection was synchronised by centrifugation at 400 x g for 30 min, followed by incubation for 2 h at 37 °C with 5% CO_2_. At 2 hpi, cells were washed three times with HBSS to remove extracellular EBs, and fresh inoculation medium was added. For transcriptomic analysis, cells were lysed at 4 and 24 hpi using Buffer SKP from the NORGEN BIOTEK DNA/RNA purification kit. Additional samples were collected at 40 hpi and fixed with methanol (Merck) for subsequent immunofluorescent analysis using the Pathfinder anti-chlamydial LPS immunofluorescent staining (**Figure 1**). Prior to all sample collection timepoints, cells were washed three times with phosphate-buffered saline (Gibco) to remove dead or unattached cells.

### Library preparation and sequencing

Cell lysates collected at 4 and 24 hpi were subjected to RNA extraction using the NORGEN BIOTEK DNA/RNA Purification Kit according to the manufacturer’s protocols for total RNA purification from cellular lysates.

For RNA sequencing, cDNA library preparation was performed using the Lexogen QuantSeq 3’ mRNA-Seq Library Prep Kit for Illumina (FWD) following the manufacturer’s protocol. Double-stranded cDNA libraries were purified using magnetic bead-based purification. To optimise amplification efficiency, the number of PCR cycles for library amplification was determined through quantitative PCR using the Lexogen PCR Add-on Kit for Illumina. Sample-specific barcoding was achieved using the Lexogen UDI 12 nt Unique Dual Indexing Add-on Kit to incorporate unique dual indices. Following final magnetic bead purification, libraries were quantified and pooled in equimolar ratios to ensure balanced representation during multiplexed sequencing. Pooled libraries were submitted to Lexogen for single-end sequencing on the NextSeq 2000 platform (Illumina).

### Data processing and differential expression analysis

Raw single-end sequencing reads were processed through a standardised bioinformatics pipeline. Adapter trimming and quality filtering were performed using Cutadapt (version 1.18), followed by quality assessment using FastQC (v0.11.7). Trimmed reads were aligned to the human reference genome (GRCh38, Ensembl release 107) using STAR aligner (v2.6.1a), and gene-level read quantification was performed using featureCounts (v1.6.4). Alignment quality metrics, including the percentage of uniquely mapped reads, were compiled using MultiQC (v1.5).

Differential gene expression analysis was conducted in R (v3.6.0) using RStudio (v2024.04.02) with the tidyverse package (v1.2.1) for data manipulation and visualisation. To ensure robust statistical analysis, genes with insufficient read coverage (present in fewer than 3 samples with at least 10 counts) were filtered prior to analysis.

Individual gene differential expression testing was performed using the DESeq2 package (v1.18.1) following the standard workflow, with count data normalisation performed using DESeq2’s median-of-ratios method, which calculates sample-specific size factors by taking the median ratio of each sample’s counts to the geometric mean across all samples for each gene to account for differences in sequencing depth and RNA composition. Genes were classified as differentially expressed if they met the criteria of log2 Fold Change (log2FC) > 1 and adjusted *P*-value < 0.05 (Benjamini-Hochberg multiple testing correction (BHmtc)).

Statistical comparison of DEG counts between experimental conditions was performed using Fisher’s exact test to determine whether the number of DEGs differed significantly between treatment groups, cell lines, time points, and bacterial strains. Ensembl gene identifiers were converted to HGNC gene symbols using the org.Hs.eg.db annotation package (v3.8.2).

### Data visualisation and exploratory analysis

Principal component analysis (PCA) was performed to visualise sample clustering and assess global transcriptional patterns across experimental conditions. Normalised count data for differentially expressed genes were subjected to z-score scaling (mean-centred and scaled to unit variance) to standardise gene expression values across samples prior to dimensionality reduction. PCA was prepared using the prcomp() function in R. Sample clustering was visualised using ggplot2 (v3.3.2), and confidence ellipses drawn around each group using the ggforce package (v0.3.2) to highlight cluster boundaries. Sample labels were added using the ggrepel (v0.9.6), and a custom colour palette was generated using RColorBrewer (v1.1.3).

Gene expression heatmaps were generated to visualise hierarchical clustering patterns and identify co-expressed gene modules across experimental conditions using the pheatmap package (v1.0.12). Normalised count data were partitioned into experimental groups and subsequently merged and subjected to z-score scaling (mean-centred and scaled to unit variance) to standardise expression values and enable comparison across genes with different baseline expression levels. Hierarchical clustering was performed using Euclidean distance metrics. Dendrograms were cut to form 4 distinct clusters for both genes and samples to identify major expression patterns, with a custom colour palette.

### Pathway enrichment analysis

Functional enrichment analysis was performed to identify biological pathways and processes associated with differentially expressed genes using Gene Set Enrichment Analysis (GSEA) with the clusterProfiler (v4.12.6), fgsea (v1.30.0), and org.Hs.eg.db packages in R. For Gene Ontology (GO) Biological Process (BP) analysis, genes were ranked based on log2FC values to preserve the magnitude and direction of expression changes across the entire transcriptome. GO BP and associated gene sets were retrieved from the org.Hs.eg.db annotation database using the AnnotationDbi::select() function from the AnnotationDbi package (v1.66.0), with analysis restricted to gene sets containing 15–1000 genes to ensure statistical power whilst excluding overly broad or highly specific categories. GSEA was conducted using the gseGO() function from clusterProfiler (v4.12.6), focusing on BP ontology terms with a significance threshold of adjusted *P*-value < 0.05 using BHmtc. To reduce redundancy amongst significantly enriched GO BP, pathway consolidation was performed using the collapsePathways function from fgsea, which retains the most representative pathways from clusters of overlapping gene sets based on pathway similarity.

For KEGG pathway analysis, HGNC gene symbols were converted to ENTREZ identifiers using the bitr() function from clusterProfiler, with genes lacking valid ENTREZ IDs or duplicate mappings removed to maintain analytical integrity. Pre-ranked gene lists were analysed using the gseKEGG() function against the human KEGG database (organism = “hsa”) with identical gene set size restrictions (15–1000 genes). For KEGG analyses, pathways with adjusted *P*-values < 0.05 (BHmtc) were considered statistically significant. Pathways were classified as upregulated or downregulated based on their normalised enrichment scores. Significantly enriched pathways were grouped into biological domains based on interpretation of pathway annotations from GO and KEGG databases.

### Pathway visualisation and comparative analysis

Enriched GO BP and KEGG pathway data were visualised using customised combined bar and dot plots generated with ggplot2. Gene ratios and adjusted *P*-values were processed to create bar plots displaying enrichment scores. Network plots were generated using the cnetplot() function from clusterProfiler to visualise relationships between significantly enriched pathways and their constituent genes, with colour gradients representing log2FC. Plot customisation was achieved using ggplot2 and gridExtra (v2.3) packages.

To identify pathway enrichment patterns across treatment conditions, intersection analysis was performed using Venn diagrams generated with the ggvenn (v0.1.10) package. Pathways were systematically categorised into four functional groups based on inoculation conditions: (*i*) CtA-specific pathways (unique to live CtA or shared between live and HIA CtA), (*ii*) CtB-specific pathways (unique to live CtB or shared between live and HIA CtB), (*iii*) core pathways (shared amongst live CtA and live CtB; live CtA and HIA CtB; live CtA, and HIA CtA and live CtB; live CtB and HIA CtA; live CtB, HIA CtB and live CtA) and (*iv*) HIA pathways (specific to HIA CtA, HIA CtB, or shared between both HIA CtA and HIA CtB).

### Pathway-associated gene expression analysis

To characterise gene-level transcriptional responses within GSEA-enriched pathways, genes belonging to significantly enriched GO BP and KEGG pathways (adjusted *P* < 0.05) were identified. This step was distinct from pathway identification, which was performed using GSEA on the complete ranked transcriptome without gene-level filtering. For comparative analysis of strain-specific expression patterns, genes were subsequently filtered to those exhibiting statistically significant differential expression (adjusted *P* < 0.05, BHmtc) in at least one strain, to ensure that response pattern classification and effect size estimation were based on reliable differential expression estimates. Expression patterns were then characterised based on the difference in log2FC magnitude between strains, with a ≥1.5-fold difference (Log2FC difference ≥ 0.585) used as the threshold to define differential strain-specific expression patterns.

Genes were categorised into six response patterns: (*i*) CtA-specific - significant only in CtA (adjusted *P* < 0.05); (*ii*) CtB-specific - significant only in CtB (adjusted *P* < 0.05); (*iii*) Concordant - significant in at least one strain with the same regulatory direction and fold-change difference < 1.5; (*iv*) CtA-dominant - significant in at least one strain with the same regulatory direction and CtA exhibiting ≥1.5 fold change; (*v*) CtB-dominant - significant in at least one strain with the same regulatory direction and CtB exhibiting ≥1.5 fold change; (*vi*) Opposite - significant in at least one strain with opposing regulatory directions (both strains showing ≥1.5 fold change). Effect sizes were calculated as Cohen’s d using the approximation d = Log2FC/lfcSE, where lfcSE represents the standard error of the Log2FC from DESeq2 output. Mean effect size for comparative analysis was calculated as the average of absolute Cohen’s d values: (Cohen’s d_CtA + Cohen’s d_CtB)/2.

Data visualisation included hierarchical clustering heatmaps generated using pheatmap (v1.0.12) with correlation-based distance metrics and Ward.D2 clustering method. Two heatmaps were generated: (*i*) all pathway-associated genes to provide comprehensive overview, and (*ii*) only significantly differentially expressed genes (adjusted *P* < 0.05 in CtA, CtB, or both) to focus on strain-specific responses. Heatmaps displayed mean normalised expression values across four conditions: CtA control, CtA infected, CtB control, and CtB infected, with row annotations indicating pathway origin and significance levels.

### Multiplex cytokine quantification

Concentrations of IL-6, CXCL10/IP-10, CCL5/RANTES, and tumor necrosis factor (TNF)-α were quantified simultaneously in cell culture supernatants using the MILLIPLEX Human Cytokine/Chemokine Magnetic Bead Panel (Merck Millipore) on a MAGPIX multiplexing instrument (Luminex). Samples (50 μL per well) were assayed undiluted and processed in accordance with the manufacturer’s instructions. Pairwise comparisons between each infection condition and the uninfected control were performed using Welch’s two-sample t-test, with *P* values adjusted for BHmtc.

## Results

### Ct infection and RNA-seq data quality assessment

HCjE and THP-1 cells were infected with Ct strains A/2497 and B/Tunis864, and confirmed by microscopy (**Supplementary Figures 1**, **2**). RNA-seq data quality metrics before and after trimming are summarised in **Supplementary Tables 1**, **2**, and **Supplementary Figure 3**. Following quality trimming and alignment using STAR, we obtained an average of 2,809,517 ± 812,089 (mean ± SD) uniquely mapped reads per sample, corresponding to 11,674 ± 1,909 detected genes. Detailed alignment statistics, including the number and percentage of uniquely mapped reads, are provided in **Supplementary Table 3** and **Supplementary Figure 4**.

### Transcriptomic responses in HCjE and THP-1 cells following Ct infection

**Figure 2:**
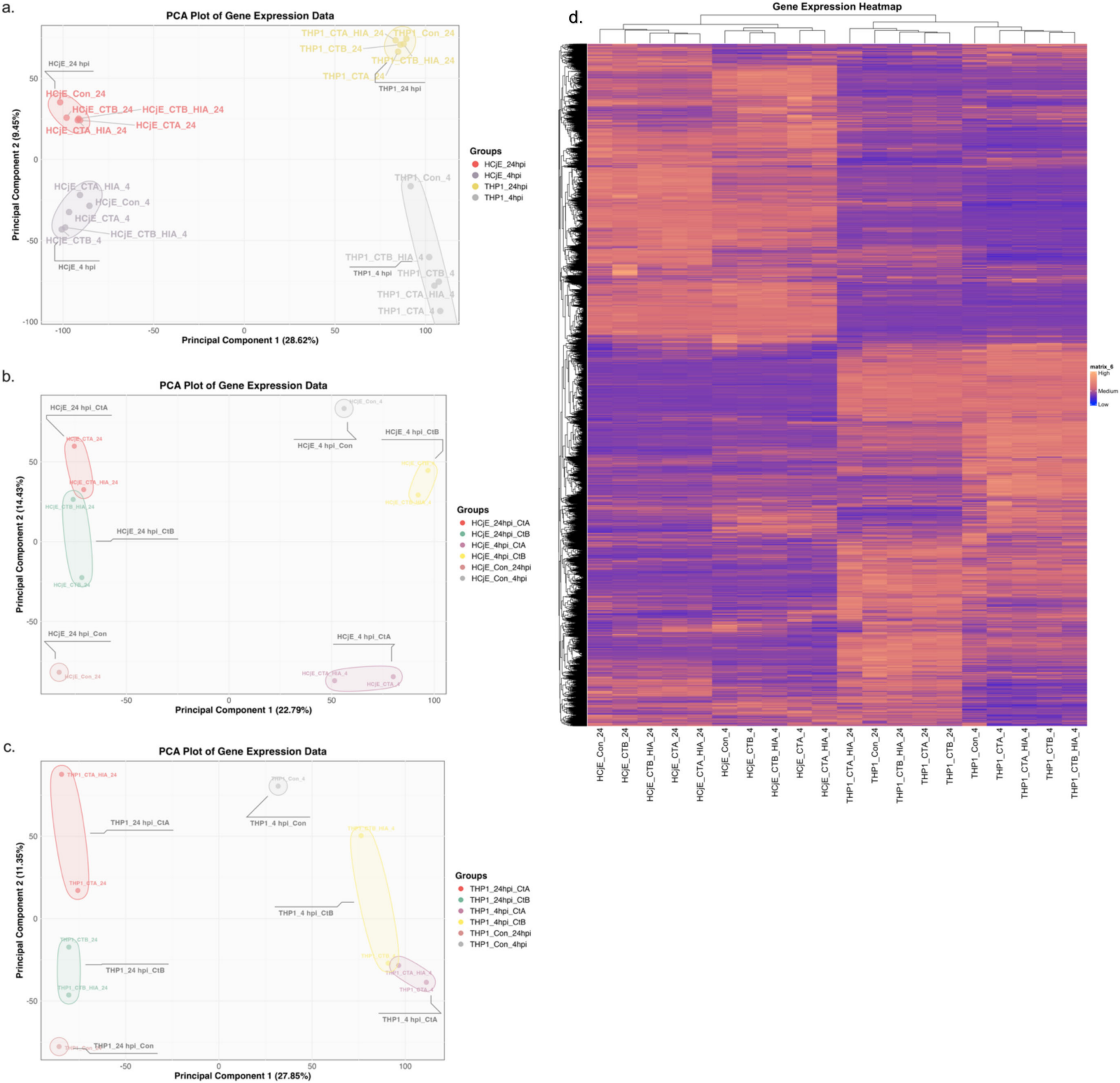
PCA and hierarchical clustering of gene expression data. **(a)** PCA plot showing sample separation by cell type (HCjE *vs*. THP-1) and time point (4 *vs*. 24 hpi) along PC1 and PC2. **(b)** PCA plot for HCjE cells demonstrating separation by time point and inoculation condition. **(c)** PCA plot for THP-1 cells showing similar separation by time point and inoculation condition. **(d)** Heatmap of normalised expression levels for 12,701 genes across all conditions. Data were normalised to the mean expression across samples, with hierarchical clustering applied to both genes (rows) and samples (columns) using complete linkage and Euclidean distance.

PCA of all experimental conditions revealed that PC1 and PC2 explained 28.6% and 9.4% of the total variance, respectively, primarily capturing variation associated with (*i*) cell type and (*ii*) time post-inoculation (**Figure 2a**). Hierarchical clustering analysis, based on normalised mean counts for each condition, supported these findings, resulting in clustering by cell line and inoculation time point (**Figure 2d**). Cell line-specific PCA showed that PC1 and PC2 accounted for 22.8% and 14.4% of variance in HCjE cells, and 27.8% and 11.3% in THP-1 cells, respectively. These components appeared to reflect differences in inoculation time point and inoculation condition (**Figures 2b, c**).

In HCjE cells, DEG numbers were significantly higher at 4 hpi compared to 24 hpi (mean DEGs: 144 *vs*. 100; *P* = 0.0458) (**Supplementary Figure 5a**). THP-1 cells showed a significant reduction in the number of DEGs from 4 hpi to 24 hpi (mean DEGs: 154 *vs*. 391; *P* < 0.0001) (**Supplementary Figure 5b**). In HCjE cells infected with A/2497 there were significantly fewer DEGs than B/Tunis864 at both time points (4 hpi: 54 *vs*. 152, *P* = 0.0003; 24 hpi: 83 *vs*. 259, *P* < 0.0001) (**Supplementary Figure 5a**). Conversely, in THP-1 cells, A/2497 infection resulted in significantly more DEGs than B/Tunis864 at both time points (4 hpi: 599 *vs*. 376, *P* < 0.0001; 24 hpi: 166 *vs*. 114, *P* = 0.0221) (**Supplementary Figure 5b**).

Analysis of DEG intersections across the eight inoculation conditions within each cell line revealed distinct patterns (**Supplementary Figure 5**). Of the total DEGs identified (HCjE: 414 at 4 hpi, 329 at 24 hpi; THP-1: 794 at 4 hpi, 369 at 24 hpi), THP-1 cells showed a significantly higher proportion of overlapping genes at each time point (4 hpi: 50.1%; 24 hpi: 36.9%) compared to HCjE cells (4 hpi: 24.6%; 24 hpi: 14%) (both *P* < 0.0001) (**Supplementary Figure 5**). The highest numbers of unique DEGs were observed in HCjE cells infected with live B/Tunis864 at 24 hpi (29.1%) and THP-1 cells infected with live A/2497 at 4 hpi (20.7%) (**Supplementary Figure 5**).

### Pathway enrichment analysis in HCjE and THP-1 cells

**Figure 3:**
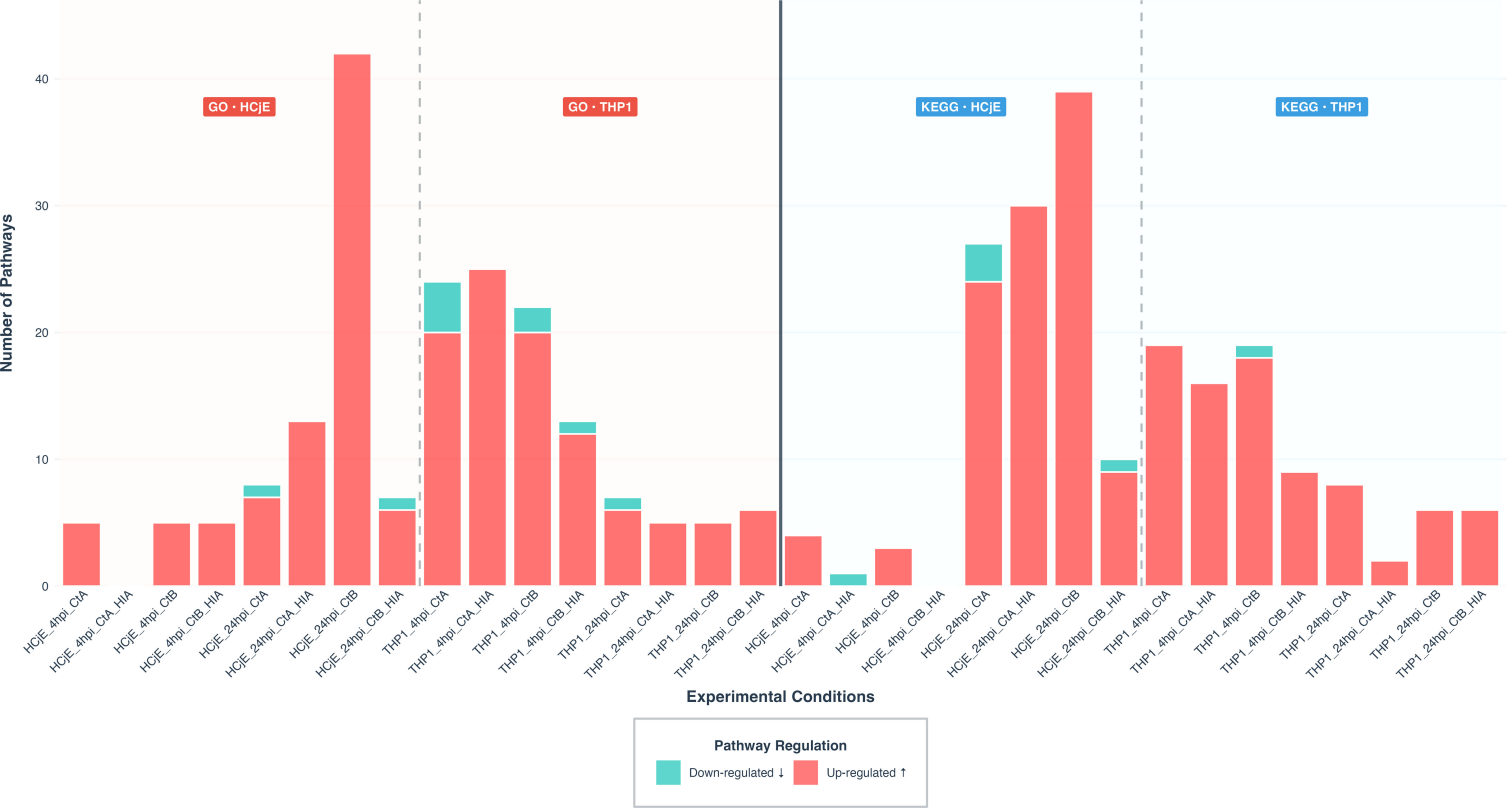
Bar plots showing the number of enriched GO BP **(a)** and KEGG **(b)** pathways. The bars represent the total number of enriched pathways in HCjE and THP-1 cells inoculated with CtA and CtB, either live or HIA. Upregulated pathways are shown in red, whilst downregulated pathways are shown in blue. Vertical dashed lines separate conditions associated with HCjE and THP-1 cells, whereas horizontal dashed lines group conditions based on inoculation time points.

**Figure 4:**
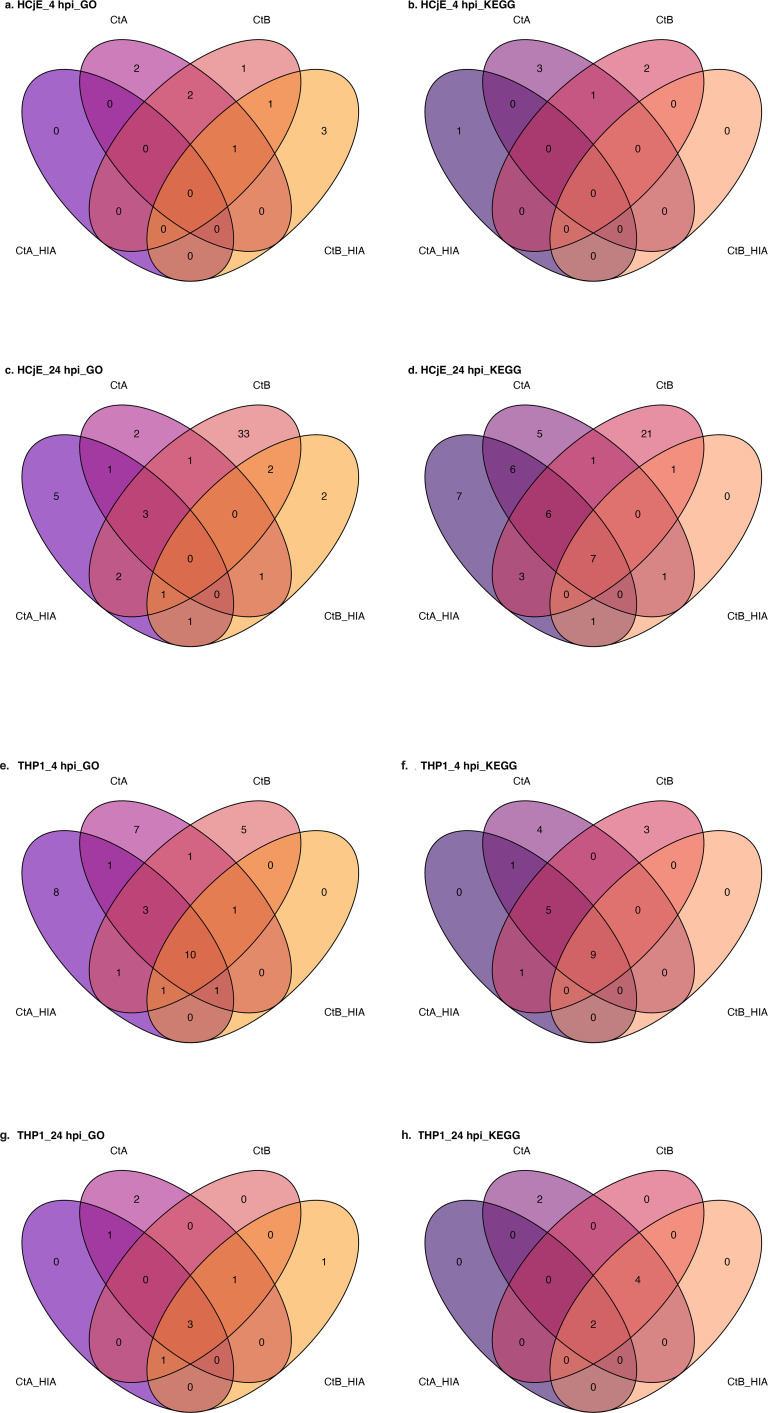
Venn diagrams showing pathway overlap between Ct inoculation conditions in HCjE and THP-1 cells. Diagrams compare GO BP and KEGG pathways identified in response to live CtA, HIA CtA, live CtB, and HIA CtB at 4 and 24 hpi. HCjE cells: GO BP pathways at 4 hpi **(a)**, KEGG pathways at 4 hpi **(b)**, GO BP pathways at 24 hpi **(c)**, KEGG pathways at 24 hpi **(d)**. THP-1 cells: GO BP pathways at 4 hpi **(e)**, KEGG pathways at 4 hpi **(f)**, GO BP pathways at 24 hpi **(g)**, KEGG pathways at 24 hpi **(h)**.

There was an increase in the mean number of enriched pathways from 4 hpi to 24 hpi for both GO BP (2.5 to 13.5) and KEGG (1.75 to 14.75) in HCjE cells (**Figure 3**). At 4 hpi, 10 GO BP and 7 KEGG pathways were enriched, with no pathways shared across all inoculation conditions (**Figures 4a, b**). By 24 hpi, pathway enrichment had increased to 54 GO BP and 59 KEGG pathways (**Figure 3**) out of which no GO BP and 7 KEGG pathways (11.9%) were conserved across all inoculation conditions (**Figures 4c, d**). At 24 hpi, live B/Tunis864 infection resulted in higher number of differentially enriched GO BP and KEGG pathways than live A/2497 infection (GO BP: 42 *vs*. 8 and KEGG: 39 *vs.* 27) (**Figure 3**).

In contrast to HCjE cells, THP-1 cells exhibited a decrease in pathway enrichment from 4 hpi to 24 hpi for both GO BP (9.75 to 2.25) and KEGG (5.75 to 2) analyses (**Figure 3**). At 4 hpi, 39 GO BP and 23 KEGG pathways were enriched, with overlap across conditions: 10 GO BP pathways (25.6%) and 9 KEGG pathways (39.1%) were shared across all conditions (**Figures 4e, f**). By 24 hpi, pathway enrichment had reduced to 9 GO BP and 8 KEGG pathways, with 3 GO BP pathways (33.3%) and 2 KEGG pathways (25%) conserved across all conditions (**Figures 4g, h**).

### Pathway responses in HCjE cells

**Figure 5:**
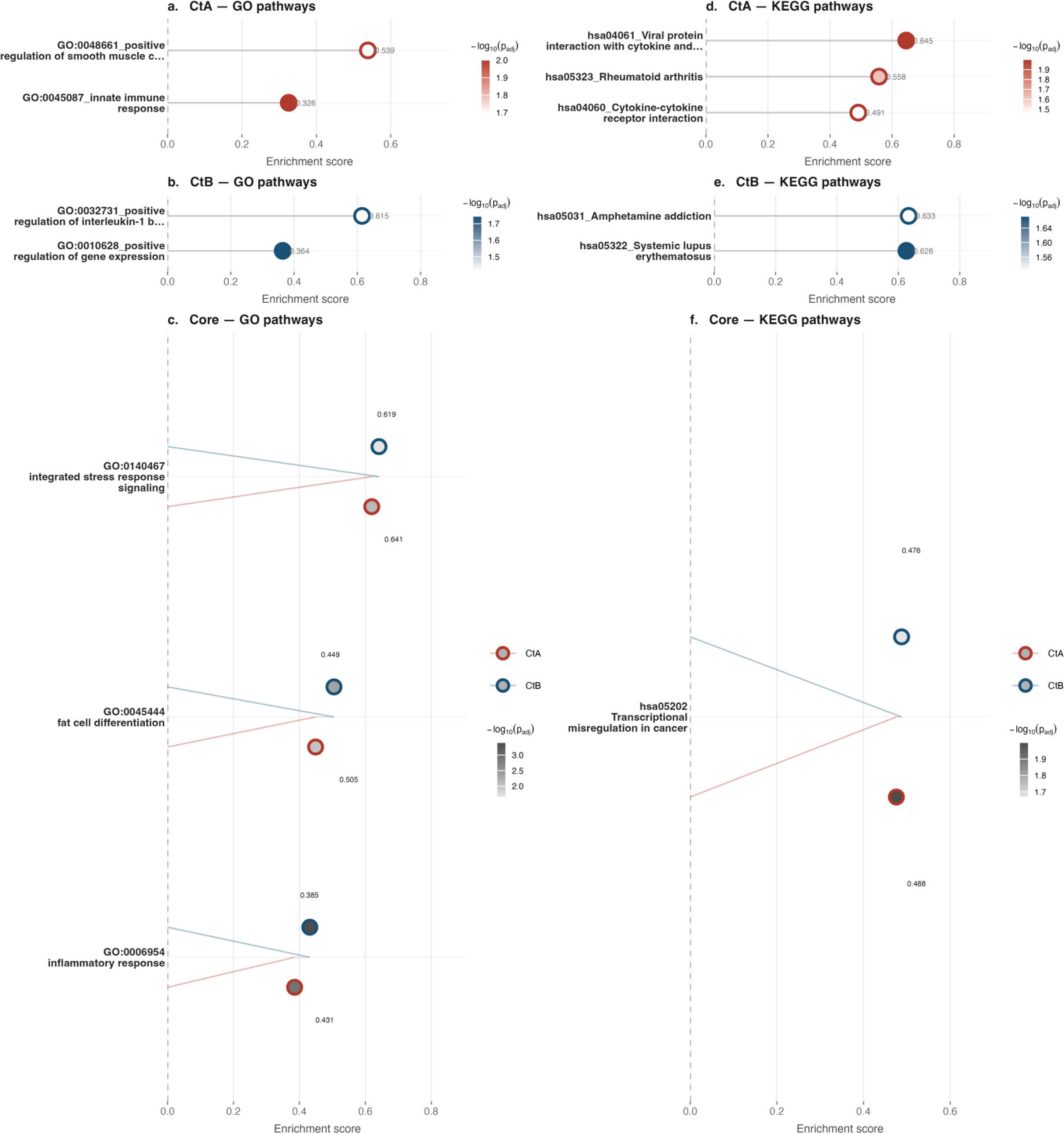
GSEA pathway enrichment in HCjE cells at 4 hpi. Lollipop dot plots of the top 10 enriched GO (left, panels **a–c**) and KEGG (right, panels **d–f**) pathways. Panels a/d and b/e show CtA- and CtB-specific pathways, respectively; panels c/f show core pathways. Dot fill intensity reflects statistical significance (-log10 adjusted p-value); border colour indicates enrichment direction in single-condition panels (crimson: positive; purple: negative), and infection condition in common pathway panels (CtA: crimson; CtB: navy; CtA-HIA: orange; CtB-HIA: teal). Numerical enrichment scores are shown at each dot.

At 4 hpi in HCjE cells, pathway enrichment analysis revealed distinct strain-specific responses alongside shared core pathways (**Figures 4a, b**, **5a-f**). Both strains upregulated core pathways involved in stress signalling (GO:0140467), metabolic reprogramming (GO:0045444), inflammation (GO:0006954), and transcriptional dysregulation (hsa05202). B/Tunis864 consistently exhibited higher enrichment scores across these pathways compared to A/2497 (**Figures 5c, f**, **Supplementary Figures 6a-d**, **Supplementary Table 4**).

Each strain induced two unique GO BP pathways, whilst, A/2497 uniquely enriched three KEGG pathways and B/Tunis864 enriched two (**Figures 4**, **5**, **Supplementary Table 4**). There were differences in the pathways activated through each Ct strain: A/2497 activated immune signalling pathways, including innate immune response (GO:0045087), cytokine signalling (hsa04060 and hsa04061), and tissue remodelling (GO:0048661) (**Figures 5a, d**, **Supplementary Figures 6e, g**, **Supplementary Table 4**). In contrast, B/Tunis864 induced more targeted inflammatory responses, notably IL-1 beta production (GO:0032731), autoimmune-like mechanisms (hsa05322), and neuro-immune interactions (hsa05031) (**Figures 5b, e**, **Supplementary Figures 6f, h**, **Supplementary Table 4**).

**Figure 6:**
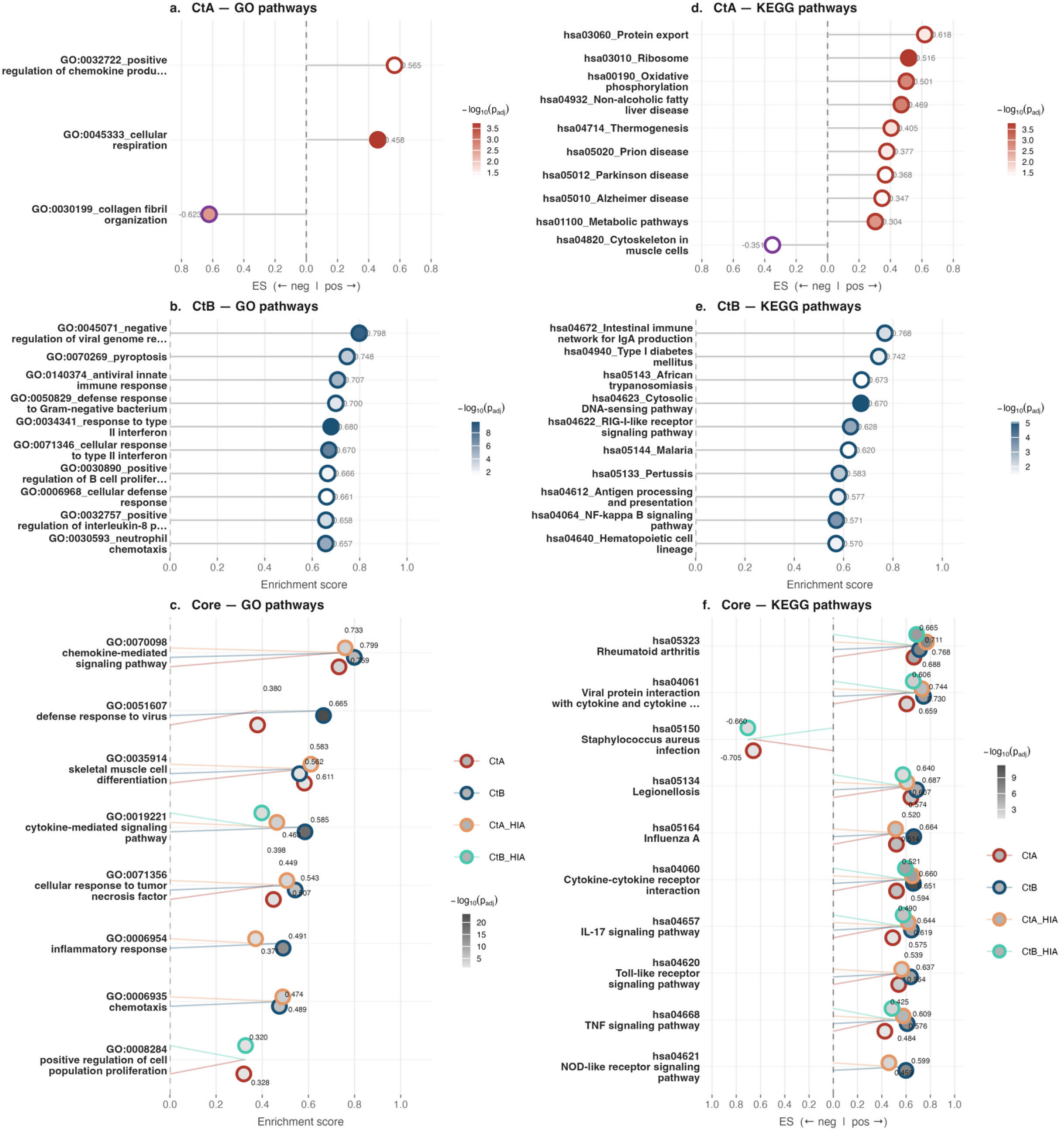
GSEA pathway enrichment in HCjE cells at 24 hpi. Lollipop dot plots of the top 10 enriched GO (left, panels **a–c**) and KEGG (right, panels **d–f**) pathways. Panels a/d and b/e show CtA- and CtB-specific pathways, respectively; panels c/f show core pathways. Dot fill intensity reflects statistical significance (-log10 adjusted p-value); border colour indicates enrichment direction in single-condition panels (crimson: positive; purple: negative), and infection condition in common pathway panels (CtA: crimson; CtB: navy; CtA-HIA: orange; CtB-HIA: teal). Numerical enrichment scores are shown at each dot.

By 24 hpi, pathway enrichment had diverged markedly between strains. A/2497 uniquely enriched two GO BP pathways compared to 35 for B/Tunis864, whilst eight GO BP pathways remained commonly enriched (**Figures 4c**, **6a-c**, **Supplementary Table 5**). For KEGG, A/2497 enriched 11 unique pathways, B/Tunis864 enriched 22, and 18 were shared between strains (**Figures 4d**, **6d-f**, **Supplementary Table 5**). The shared core pathways encompassed three main biological domains: (*i*) immune and inflammatory responses, including cytokine-cytokine receptor interaction (hsa04060), chemokine-mediated signalling (GO:00700989), and Toll-like receptor (TLR) signalling (hsa04620); (*ii*) pathogen-host interactions, encompassing viral protein interactions with cytokine receptors (hsa04061) and antiviral defence responses (GO:0051607); and (*iii*) cellular stress and pathological processes, including lipid metabolism in atherosclerosis (hsa05417) and transcriptional dysregulation in cancer (hsa05202). Across all shared pathways, B/Tunis864 maintained consistently higher enrichment scores than A/2497 (**Figures 6c, f**, **Supplementary Table 5**).

At 24 hpi, A/2497 engaged pathways involved in cellular maintenance and energy metabolism including oxidative phosphorylation (hsa00190), ribosomal function (hsa03010), and protein export machinery (hsa03060), suggesting a focus on basic cellular homeostasis (**Figure 6d**, **Supplementary Figures 7e, g**, **Supplementary Table 5**). In contrast, B/Tunis864 activated pathways related to immune response, spanning both innate and adaptive immunity including intestinal IgA production networks (hsa04672), antigen processing and presentation (hsa04612), NF-κB signalling (hsa04064), JAK-STAT signalling (hsa04630), antiviral innate immunity (GO:0140374), cytokine production regulation (GO:0001819), anti-bacterial defence responses (GO:0050829), and TNF-mediated signalling (GO:0033209). Additionally, B/Tunis864 triggered cellular stress and death pathways, including apoptosis (hsa04210) and ERK1/ERK2 cascade activation (GO:0070374) (**Figures 6b, e**, **Supplementary Figures 7f, h**, **Supplementary Table 5**).

### Pathway responses in THP-1 cells

**Figure 7:**
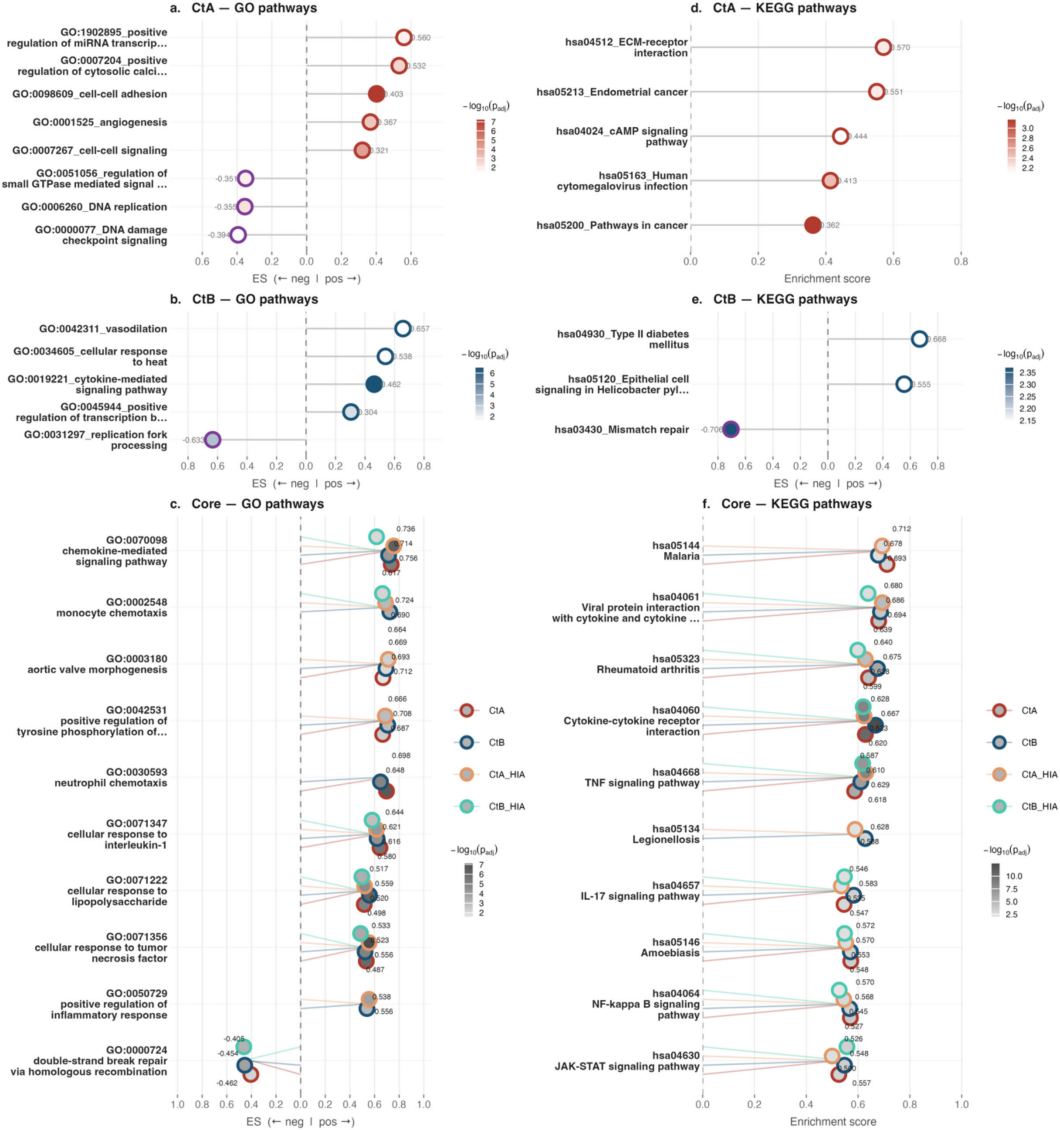
GSEA pathway enrichment in THP1 cells at 4 hpi. Lollipop dot plots of the top 10 enriched GO (left, panels **a–c**) and KEGG (right, panels **d–f**) pathways. Panels a/d and b/e show CtA- and CtB-specific pathways, respectively; panels c/f show core pathways. Dot fill intensity reflects statistical significance (-log10 adjusted p-value); border colour indicates enrichment direction in single-condition panels (crimson: positive; purple: negative), and infection condition in common pathway panels (CtA: crimson; CtB: navy; CtA-HIA: orange; CtB-HIA: teal). Numerical enrichment scores are shown at each dot.

At 4 hpi in THP-1 cells, pathway enrichment analysis revealed predominantly shared responses between strains, with 18 GO BP pathways commonly enriched by both strains, whilst A/2497 uniquely enriched 8 pathways and B/Tunis864 enriched 5 pathways (**Figures 4e**, **7a-c**, **Supplementary Table 6**). Amongst KEGG pathways, 15 were shared between strains, with 5 unique to A/2497 and 3 to B/Tunis864 (**Figure 4f**, **7d-f**, **Supplementary Table 6**). The shared core pathways encompassed three main biological domains: (*i*) immune and inflammatory responses, encompassing TNF signalling (hsa04668), inflammatory response (GO:0006954), chemokine-mediated signalling (GO:0070098), and NF-κB signalling (hsa04064); and (*ii*) pathogen-host interactions and cellular stress responses, including viral protein interactions with cytokine receptors (hsa04061), Notch signalling (GO:0007219), and homologous recombination-mediated DNA repair (GO:0000724) (**Figures 7c, f**, **Supplementary Figures 8a-d**, **Supplementary Table 6**). Notably, both strains triggered pathways involved in DNA regulation and repair processes.

At 4 hpi, A/2497 specifically triggered DNA replication machinery (GO:0006260) and DNA damage checkpoint signalling (GO:0000077), alongside cellular communication pathways including cell-cell signalling (GO:0007267) and cAMP signalling (hsa04024) (**Figures 7a, d**, **Supplementary Figures 8e, g**, **Supplementary Table 6**). In contrast, B/Tunis864 triggered DNA mismatch repair (hsa03430) and replication fork processing (GO:0031297) pathways (**Figures 7b, e**, **Supplementary Figure 8f, h**, **Supplementary Table 6**).

**Figure 8:**
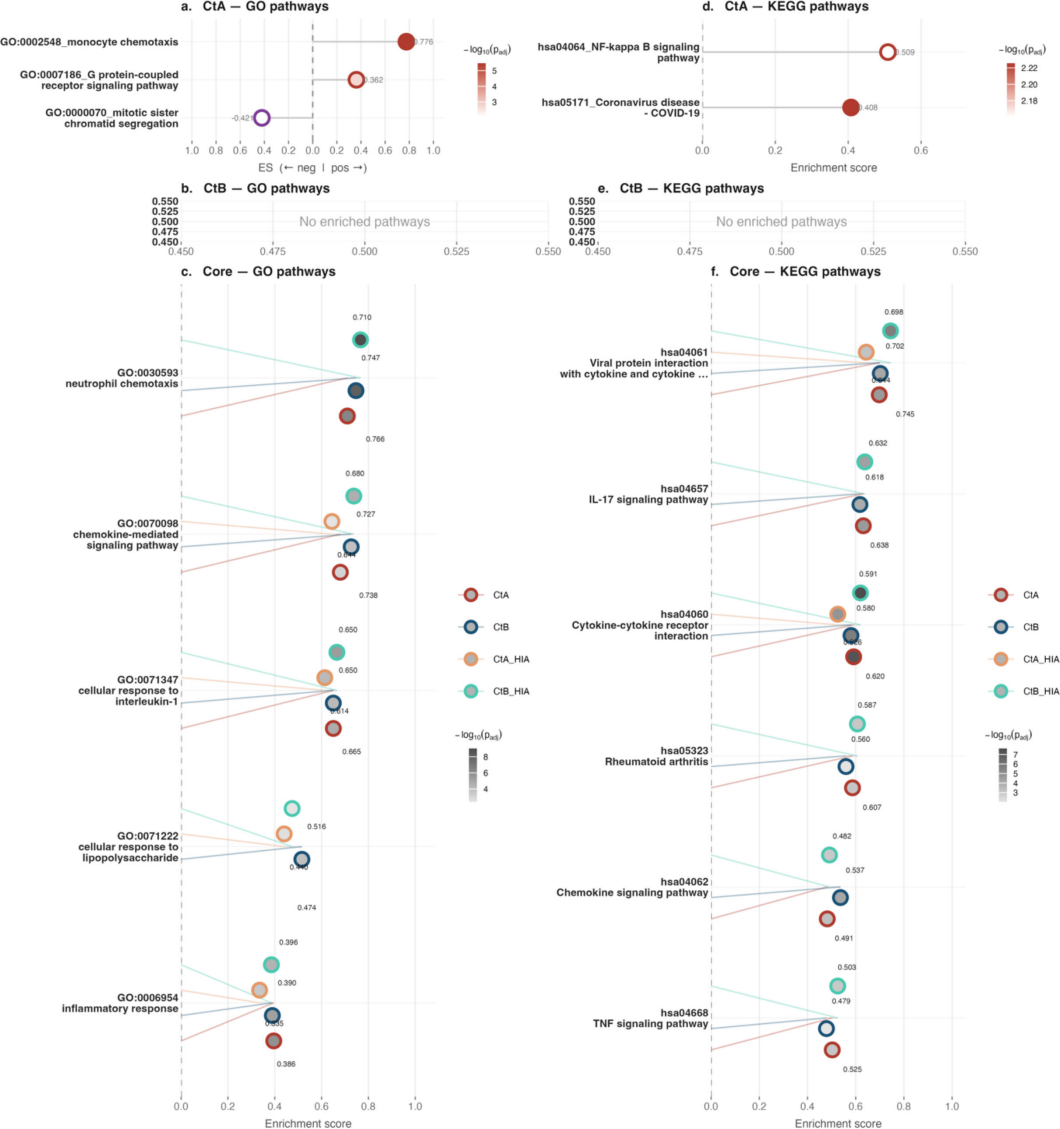
GSEA pathway enrichment in THP-1 cells at 24 hpi. Lollipop dot plots of the top 10 enriched GO (left, panels **a–c**) and KEGG (right, panels **d–f**) pathways. Panels a/d and b/e show CtA- and CtB-specific pathways, respectively; panels c/f show core pathways. Dot fill intensity reflects statistical significance (-log10 adjusted p-value); border colour indicates enrichment direction in single-condition panels (crimson: positive; purple: negative), and infection condition in common pathway panels (CtA: crimson; CtB: navy; CtA-HIA: orange; CtB-HIA: teal). Numerical enrichment scores are shown at each dot.

By 24 hpi, pathway enrichment had diminished, with only 5 GO BP pathways commonly enriched by both strains and 3 unique to A/2497, whilst no GO BP pathways were identified specifically for B/Tunis864 (**Figure 4g**, **8a-c**, **Supplementary Table 7**). Amongst KEGG pathways, 6 remained shared between strains, 2 were unique to A/2497, and no pathways were specific to B/Tunis864 (**Figure 4h**, **8d-f**, **Supplementary Table 7**). The shared pathways at 24 hpi were predominantly inflammatory and tissue-damaging responses, including TNF signalling (hsa04668), neutrophil chemotaxis (GO:0030593), inflammatory response (GO:0006954), chemokine-mediated signalling (GO:0070098), and rheumatoid arthritis-associated pathways (hsa05323) (**Figures 8c, f**, **Supplementary Figures 9a-d**, **Supplementary Table 7**). At 24 hpi, A/2497 maintained unique activation of immune-regulatory pathways, specifically monocyte chemotaxis (GO:0002548) and NF-κB signalling (hsa04064), alongside a cellular process pathway involving mitotic sister chromatid segregation (GO:0000070) (**Figures 8a, d**, **Supplementary Figures 9e, f**, **Supplementary Table 7**).

### Responses to live and HIA Ct

In HCjE cells, at 4 hpi, there was no core enriched pathways including integrated stress response signalling (GO:0140467) and inflammatory response (GO:0006954) under HIA conditions, which may indicate these early signatures are predominantly replication-dependent. By 24 hpi, the extensive IFN-stimulated gene (ISG) signature unique to live CtB, encompassing cytosolic DNA-sensing (hsa04623), RIG-I-like receptor signalling (hsa04622), and upregulation of IFIT1–3, MX1, MX2, OAS1, OAS2, CXCL10, and STAT1, was absent under HIA conditions, suggesting requirement for active RB replication. Similarly, the CtA-specific metabolic programme (hsa00190, hsa03010, hsa03060) was absent in HIA conditions. In contrast, broader immune signalling pathways including cytokine-cytokine receptor interaction (hsa04060), TNF signalling (hsa04668), and TLR signalling (hsa04620) were enriched under both live and HIA conditions, maybe suggesting these as pattern recognition receptors (PRR)-mediated responses independent of replication.

In THP-1 cells, at 4 hpi, broad overlap between live and HIA conditions across core inflammatory pathways, including TNF signalling (hsa04668), NF-κB signalling (hsa04064), cytokine-cytokine receptor interaction (hsa04060), and TLR signalling (hsa04620), may indicate that the initial macrophage response is largely replication-independent. By 24 hpi, whilst core inflammatory pathways including neutrophil chemotaxis (GO:0030593) and TNF signalling (hsa04668) remained enriched under both conditions, the CtA-specific activation of monocyte chemotaxis (GO:0002548) and NF-κB signalling (hsa04064) was detected exclusively under live infection, which may suggest that sustained replication-dependent inflammatory programming characterises live CtA infection at the mid-developmental stage.

### Pathway-associated genes in KEGG and GO BP enrichment

**Figure 9:**
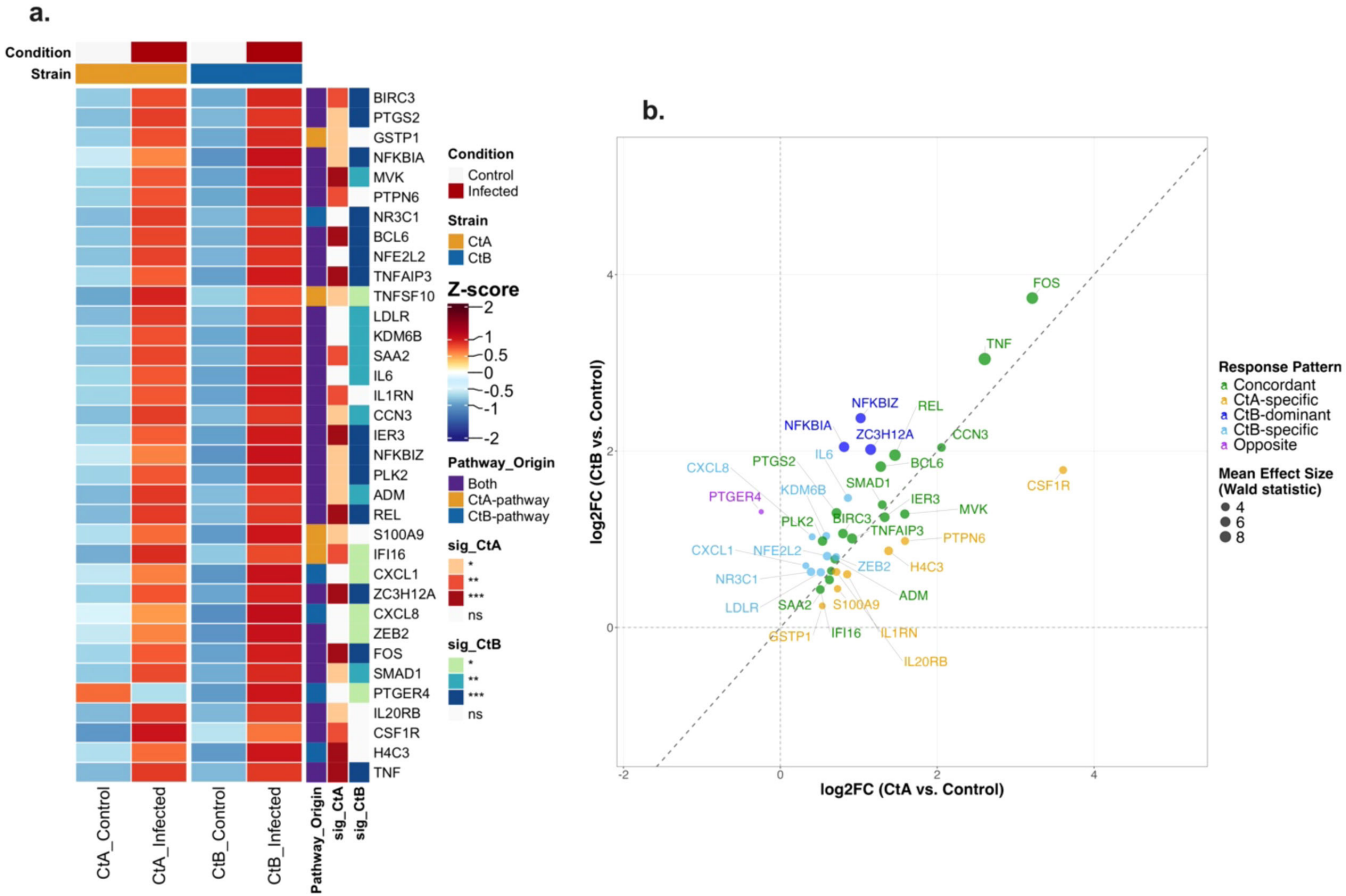
Differential expression patterns of pathway-associated genes in HCjE cells infected with Ct strains at 4 hpi. **(a)** Heatmap showing normalised expression (Z-scores) of pathway-associated genes significantly differentially expressed (adjusted *P* < 0.05) in CtA (A/2497) and/or CtB (B/Tunis864) infection (for all pathway-associated genes regardless of significance, see **Supplementary Figure 6i**). Columns: mean expression across replicates for controls and infected samples. Rows: genes ordered by pathway origin. Row annotations: pathway source (red: CtA-pathway; blue: CtB-pathway; orange: both; grey: neither) and strain-specific significance levels (***P < 0.001, **P < 0.01, *P < 0.05, white: ns). Column annotations: condition (grey: control; orange: infected) and strain (orange: CtA; blue: CtB). **(b)** Scatter plot of log2FC values comparing CtA *vs*. CtB responses for significantly differentially expressed genes. Points coloured by response pattern: orange (CtA-specific), light blue (CtB-specific), green (Concordant, < 1.5 fold change), red (CtA-dominant, ≥ 1.5 fold change), dark blue (CtB-dominant, ≥ 1.5 fold change), purple (Opposite directions). Point size reflects mean effect size (Wald statistic). Diagonal dashed line indicates equal responses; axes intersect at baseline. Top genes per response pattern are labelled.

In HCjE cells at 4 hpi, pathway enrichment analysis identified 89 and 139 genes associated with significantly enriched pathways for A/2497 and B/Tunis864 infection, respectively. Of the 181 unique pathway-associated genes examined, 35 exhibited significant differential expression relative to uninfected controls (adjusted *P* < 0.05) (**Supplementary Figure 6i**, **Figure 9a**). Expression pattern analysis revealed predominantly balanced responses between strains: 16 genes (45.7%) demonstrated concordant upregulation, whilst 7 were CtA-specific and 8 were CtB-specific. Additionally, 3 genes showed CtB-dominant responses, and 1 gene displayed opposite regulatory directions (**Figure 5b**). Notable CtA-specific inflammatory mediators included IL1RN, CSF1R, IL20RB, and S100A9. In contrast, CtB-specific/dominant genes encompassed critical NF-κB pathway regulators (NFKBIZ, NFKBIA, ZC3H12A) and pro-inflammatory mediators (IL6, CXCL1, CXCL8, NR3C1) (**Figure 9b**).

**Figure 10:**
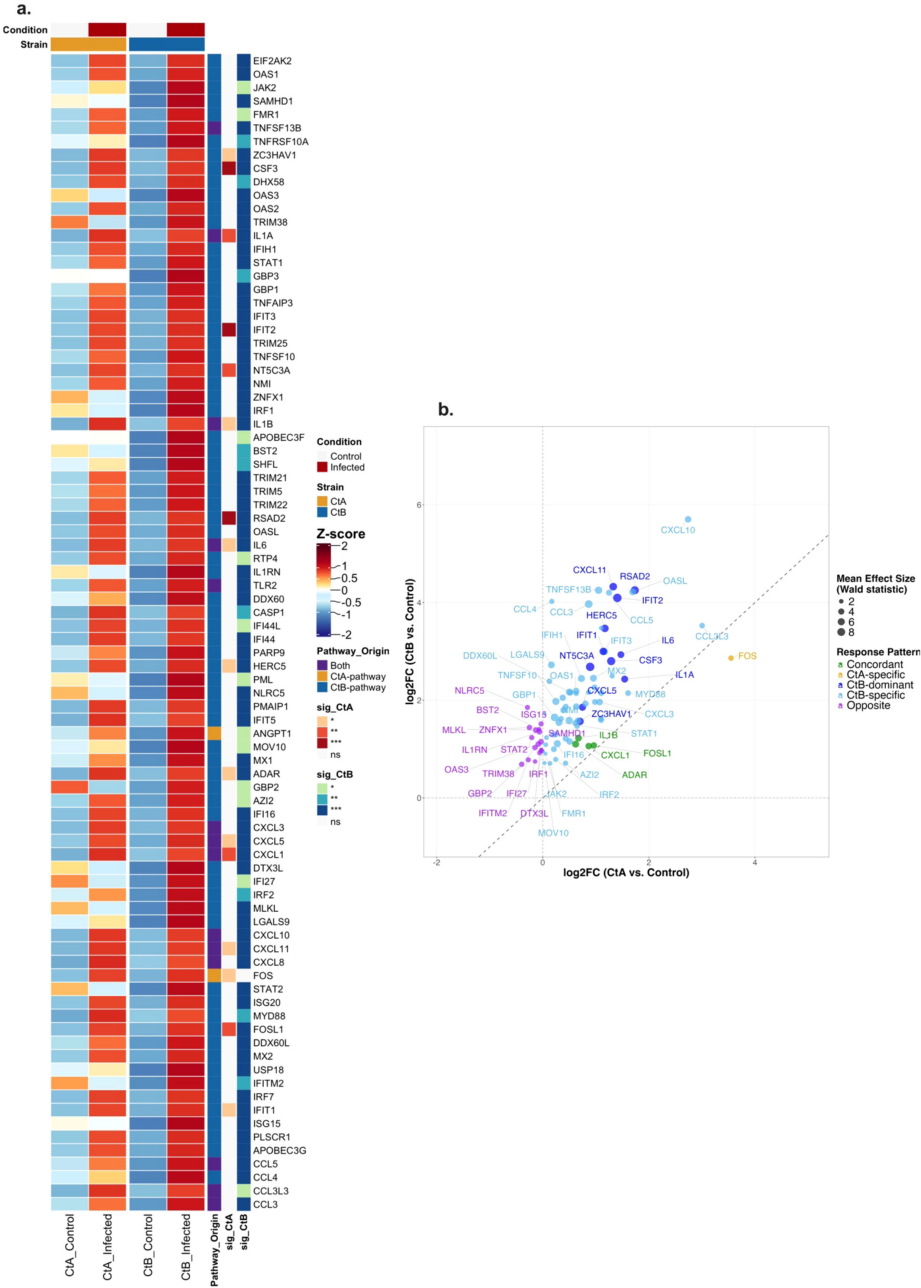
Differential expression patterns of pathway-associated genes in HCjE cells infected with Ct strains at 24 hpi. **(a)** Heatmap showing normalised expression (Z-scores) of pathway-associated genes significantly differentially expressed (adjusted *P* < 0.05) in CtA (A/2497) and/or CtB (B/Tunis864) infection (for all pathway-associated genes regardless of significance, see **Supplementary Figure 7i**). Columns: mean expression across replicates for controls and infected samples. Rows: genes ordered by pathway origin. Row annotations: pathway source (red: CtA-pathway; blue: CtB-pathway; orange: both; grey: neither) and strain-specific significance levels (***P < 0.001, **P < 0.01, *P < 0.05, white: ns). Column annotations: condition (grey: control; orange: infected) and strain (orange: CtA; blue: CtB). **(b)** Scatter plot of log2FC values comparing CtA *vs*. CtB responses for significantly differentially expressed genes. Points coloured by response pattern: orange (CtA-specific), light blue (CtB-specific), green (Concordant, < 1.5 fold change), red (CtA-dominant, ≥ 1.5 fold change), dark blue (CtB-dominant, ≥ 1.5 fold change), purple (Opposite directions). Point size reflects mean effect size (Wald statistic). Diagonal dashed line indicates equal responses; axes intersect at baseline. Top genes per response pattern are labelled.

At 24 hpi in HCjE cells, pathway enrichment analysis identified 29 and 163 genes associated with significantly enriched pathways for A/2497 and B/Tunis864 infection, respectively. Of the 172 unique pathway-associated genes examined, 86 exhibited significant differential expression (**Supplementary Figure 7i**, **Figure 10a**). Expression patterns revealed markedly strain-differentiated responses: B/Tunis864 infection induced 54 strain-specific and 11 strain-dominant genes (collectively 75.6% of significant genes), whilst A/2497 elicited only 1 strain-specific gene. Concordant responses declined to 4 genes, and 16 genes displayed opposite regulatory directions (**Figure 10b**). The B/Tunis864-dominant response was characterised by robust ISG activation, including IFIT1, IFIT2, IFIT3, HERC5, MX1, MX2, OAS1, OAS2, and RSAD2, alongside IFN-inducible chemokines (CXCL10, CXCL11) and pro-inflammatory mediators (IL1A, IL6, CCL3, CCL5, TNFSF10, TNFSF13B). STAT1, a key IFN signalling transcription factor, was also exclusively upregulated in B/Tunis864 infection. ISG15 was significantly upregulated in CtB infection whilst there were no significant changes in CtA infection further highlighting divergent immune responses between strains (**Figure 10b**).

**Figure 11:**
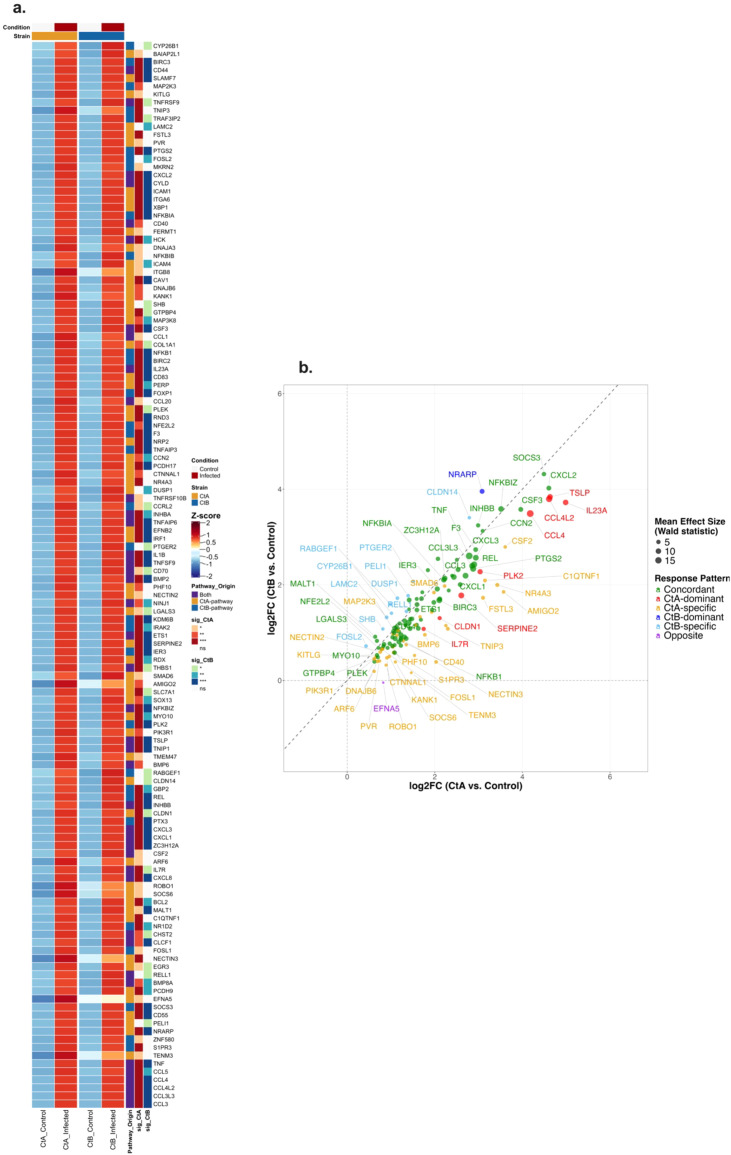
Differential expression patterns of pathway-associated genes in THP-1 cells infected with Ct strains at 4 hpi. **(a)** Heatmap showing normalised expression (Z-scores) of pathway-associated genes significantly differentially expressed (adjusted *P* < 0.05) in CtA (A/2497) and/or CtB (B/Tunis864) infection (for all pathway-associated genes regardless of significance, see **Supplementary Figure 8i**). Columns: mean expression across replicates for controls and infected samples. Rows: genes ordered by pathway origin. Row annotations: pathway source (red: CtA-pathway; blue: CtB-pathway; orange: both; grey: neither) and strain-specific significance levels (***P < 0.001, **P < 0.01, *P < 0.05, white: ns). Column annotations: condition (grey: control; orange: infected) and strain (orange: CtA; blue: CtB). **(b)** Scatter plot of log2FC values comparing CtA *vs*. CtB responses for significantly differentially expressed genes. Points coloured by response pattern: orange (CtA-specific), light blue (CtB-specific), green (Concordant, < 1.5 fold change), red (CtA-dominant, ≥ 1.5 fold change), dark blue (CtB-dominant, ≥ 1.5 fold change), purple (Opposite directions). Point size reflects mean effect size (Wald statistic). Diagonal dashed line indicates equal responses; axes intersect at baseline. Top genes per response pattern are labelled.

In THP-1 cells at 4 hpi, pathway enrichment analysis identified 225 and 136 genes associated with significantly enriched pathways for A/2497 and B/Tunis864 infection, respectively. Of the 305 unique pathway-associated genes examined, 132 exhibited significant differential expression (**Supplementary Figure 8i**, **Figure 11a**). Expression analysis revealed predominantly concordant inflammatory responses characteristic: 76 genes (57.6%) demonstrated similar upregulation patterns between strains. These included pro-inflammatory cytokines (TNF, IL1B, CSF3), NF-κB pathway components (NFKBIZ, REL, NFKBIA, NFKB1), and chemokines (CXCL1, CXCL2, CXCL3, CXCL8, CCL3, CCL3L3, CCL5). Modest strain-biased responses were observed: 36 genes were CtA-specific and 8 were CtA-dominant (including CCL4, CCL20, CSF2, TNIP3), whilst 10 genes were CtB-specific and 1 was CtB-dominant. Only 1 gene displayed opposite regulation (**Figure 11b**).

**Figure 12:**
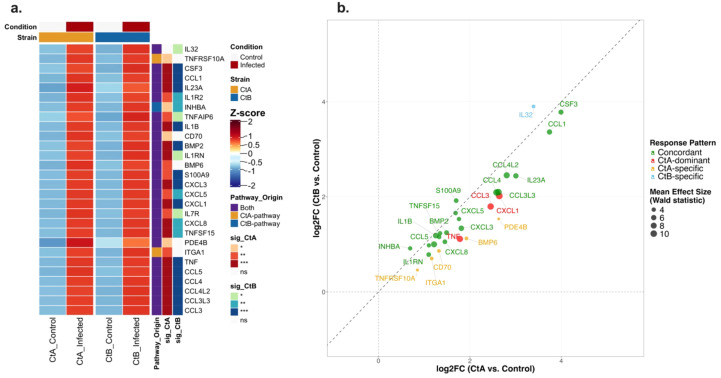
Differential expression patterns of pathway-associated genes in THP-1 cells infected with Ct strains at 24 hpi. **(a)** Heatmap showing normalised expression (Z-scores) of pathway-associated genes significantly differentially expressed (adjusted *P* < 0.05) in CtA (A/2497) and/or CtB (B/Tunis864) infection (for all pathway-associated genes regardless of significance, see **Supplementary Figure 9g**). Columns: mean expression across replicates for controls and infected samples. Rows: genes ordered by pathway origin. Row annotations: pathway source (red: CtA-pathway; blue: CtB-pathway; orange: both; grey: neither) and strain-specific significance levels (***P < 0.001, **P < 0.01, *P < 0.05, white: ns). Column annotations: condition (grey: control; orange: infected) and strain (orange: CtA; blue: CtB). **(b)** Scatter plot of log2FC values comparing CtA *vs*. CtB responses for significantly differentially expressed genes. Points coloured by response pattern: orange (CtA-specific), light blue (CtB-specific), green (Concordant, < 1.5 fold change), red (CtA-dominant, ≥ 1.5 fold change), dark blue (CtB-dominant, ≥ 1.5 fold change), purple (Opposite directions). Point size reflects mean effect size (Wald statistic). Diagonal dashed line indicates equal responses; axes intersect at baseline. Top genes per response pattern are labelled.

At 24 hpi in THP-1 cells, pathway enrichment analysis identified 51 and 45 genes associated with significantly enriched pathways for A/2497 and B/Tunis864 infection, respectively. Of the 58 unique pathway-associated genes examined, 28 exhibited significant differential expression (**Supplementary Figure 9g**, **Figure 12a**). Expression patterns revealed sustained concordant responses: 19 genes (67.9%) maintained similar upregulation between strains, demonstrating persistent inflammatory activation. Concordant genes included chemokines (CCL1, CCL3L3, CCL4, CCL4L2, CCL5, CXCL3, CXCL5, CXCL8), pro-inflammatory cytokines (IL1B, IL23A, CSF3), and TNF superfamily members (TNFSF15, TNFAIP6). Minimal strain-differentiation was observed: 5 genes were CtA-specific (including BMP6, CD70, TNFRSF10A) and 3 were CtA-dominant (TNF, CXCL1, CCL3), whilst only 1 gene was CtB-specific (IL32) (**Figure 12b**).

### Protein-level cytokine validation

**Figure 13:**
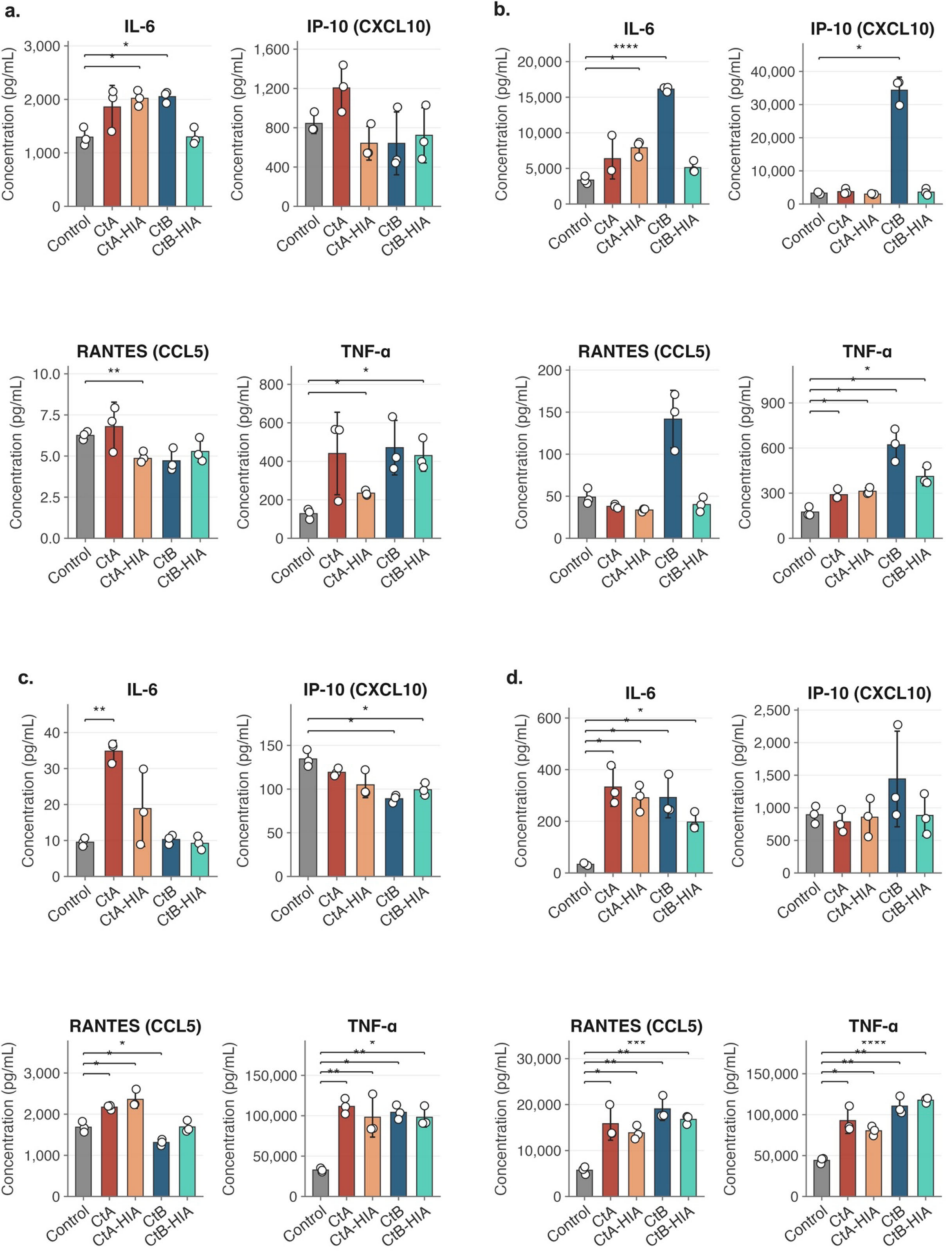
Cytokine and chemokine concentrations in HCjE and THP-1 cells following Ct infection. Concentrations of IL-6, IP-10 (CXCL10), RANTES (CCL5), and TNF-α were quantified by multiplex immunoassay in cell culture supernatants of **(a)** HCjE cells at 4 hpi, **(b)** HCjE cells at 24 hpi, **(c)** THP-1 cells at 4 hpi, and **(d)** THP-1 cells at 24 hpi. Bars represent the mean ± standard deviation of three independent biological replicates; individual data points are shown. Statistical significance was determined by Welch’s t-test against the uninfected control with Benjamini-Hochberg false discovery rate correction (*p < 0.05; **p < 0.01; ***p < 0.001; ****p < 0.0001).

To validate the transcriptomic findings at the protein level, concentrations of IL-6, CXCL10, CCL5, and TNF-α were measured (**Figure 13**). In HCjE cells, a modest but selective cytokine response was observed at 4 hpi, with notable changes of IL-6, and TNF-α concentrations in response to CtA and CtB inoculation (**Figure 13a**). By 24 hpi, the response broadened, with overall elevations in IL-6 and CXCL10 and CCL5 detected across multiple conditions. Concentrations of all four markers were notably elevated in response to CtB relative to both the uninfected control and all other inoculation conditions at this timepoint (**Figure 13b**). In THP-1 cells, TNF-α was significantly elevated across all inoculation conditions at both timepoints, whilst IL-6 and CCL5 responses were more evident at 24 hpi, with significant increases detected across all four conditions (**Figures 13c, d**). At 4 hpi, we observed higher concentration of IL-6 and CCL5 in CtA- compared to CtB- infected THP-1 cells. CXCL10 concentrations did not differ significantly from the uninfected control at either timepoint (**Figures 13c, d**).

## Discussion

In this study, we characterised the shared and distinct transcriptional responses of Ct strains A/2497 and B/Tunis864 across early- and mid-developmental stages (4 and 24 hpi) induced in HCjE and THP-1 cells. The kinetics of responses to Ct infection differed between cell types: THP-1 cells showed peak transcriptional activation at 4 hpi, whereas HCjE cells demonstrated peak transcriptional activation at 24 hpi. HCjE cells exhibited strain related transcriptional responses, contrasting with THP-1 cells that exhibited a response characterised by overlapping DEGs and enriched pathways regardless of Ct strain.

In HCjE cells, the number of DEGs and enriched pathways progressively increased from 4 to 24 hpi, consistent with established patterns of epithelial cell adaptation during chlamydial infection (Hayward et al., 2020; Dzakah et al., 2021). This temporal transcriptomic activation mirrors recent chromatin accessibility studies in Hep-2 cells, which demonstrated dramatic increases from 864 differentially accessible regions at early time points to over 3,000 regions by 48 hpi, reflecting Ct’s progressive exploitation of epithelial cellular machinery throughout its developmental cycle (Rajić et al., 2017; Hayward et al., 2019; Hayward et al., 2020). Recent studies have shown that HCjE cells support replication of major Ct genovars whilst exhibiting genovar-specific cytokine production patterns (Derrick et al., 2016; Faris et al., 2019; Smith and Valdivia, 2025), consistent with our observation of marked strain-specific transcriptional differences where CtB consistently elicited stronger responses than CtA at both time points.

Conversely, THP-1 macrophages exhibited decreasing pathway enrichment over time, consistent with the temporal dynamics of macrophage responses to intracellular pathogens (Liu et al., 2023). This pattern likely reflects the binary outcome of macrophage-*Chlamydia* interactions: either successful pathogen clearance through robust early inflammatory responses, or establishment of infection through evasion of macrophage defence mechanisms, including subversion of phagosome maturation, modulation of pro-inflammatory cytokine production, and interference with macrophage polarisation (Gracey et al., 2013; Buchacher et al., 2015; Weiss and Schaible, 2015; Yeung et al., 2017; Thakur et al., 2019; Stelzner et al., 2023; Marrufo and Flores-Mireles, 2024). Unlike the pronounced strain-specific responses observed in HCjE cells, THP-1 macrophages displayed predominantly shared responses to CtA and CtB, suggesting that macrophages deploy a core response regardless of infecting strain. This pattern of strain-invariant macrophage responses has been similarly observed in infections with *Mycobacterium abscessus* Smooth and Rough variants (Aulicino et al., 2015), suggesting that macrophages may prioritise rapid, generalised defence mechanisms over strain-specific responses during initial encounter with Ct.

At 4 hpi, HCjE cells displayed markedly different transcriptional responses to CtA and CtB infection, with CtB inducing nearly 3-fold more DEGs than CtA. Despite activating distinct strain-specific pathways, both strains upregulated core stress responses, including integrated stress response signalling (GO:0140467) and inflammatory response (GO:0006954), with CtB consistently exhibiting higher enrichment scores across these shared pathways. By 24 hpi, both the number of DEGs and enriched pathways increased substantially compared to 4 hpi, consistent with prior studies in epithelial cells demonstrating that immune response activation to Ct becomes pronounced during mid-cycle infection (6–24 hpi) (Derbigny et al., 2007; Hayward et al., 2021).

At 24 hpi, CtA activated pathways associated with cellular maintenance and metabolic homeostasis in HCjE cells, including oxidative phosphorylation (hsa00190), ribosomal function (hsa03010), and protein export machinery (hsa03060). This metabolic signature is consistent with the well-characterised capability of Ct to reprogramme host metabolism, redirecting cellular resources to support bacterial replication through glucose-6-phosphate utilisation and direct ATP scavenging via nucleotide transporters (Omsland et al., 2014; N’Gadjaga et al., 2022; Stelzner et al., 2023). Whilst certain host metabolic pathways are downregulated during chlamydial infection, including TCA cycle activity and p53 signalling (Koster et al., 2022; Cheong et al., 2025), others such as glycolysis and oxidative phosphorylation are upregulated (N’Gadjaga et al., 2022). The upregulation observed here thus may reflect the anabolic side of this metabolic hijacking programme, although differences in tissue tropism between ocular and genital strains, as well as the use of immortalised cell lines rather than primary organoid models, should be considered when interpreting the magnitude of these responses. In contrast, CtB mounted a robust immune and stress response. The extensive transcriptional activation induced by CtB in HCjE cells, particularly the pronounced ISG response at 24 hpi (CXCL10, CXCL11, MX1, OAS2, ISG20, TRIM5, TRIM22, STAT1), may explain the prior observations on more severe pathology associated with CtB than CtA in trachoma patients (Chin et al., 2018). This ISG signature reflects type-I IFN pathway activation through STING-mediated recognition of chlamydial cyclic di-AMP, a response that, whilst initially protective, may contribute to the immunopathology characteristic of trachoma when dysregulated (Lad et al., 2005; Barker et al., 2013; Wan et al., 2020; Fontanilla et al., 2024; Lee and Ouellette, 2025; Smith and Valdivia, 2025). CtB induced a pronounced type II IFN response compared to CtA. The specific mechanisms underlying this strain-specific differential response remain to be understood, though this pattern is consistent with broader *Chlamydia* immune evasion strategies that include suppression of cytokine and chemokine production by epithelial cells (Buckner et al., 2013; Wang et al., 2024), and interference with antigen presentation through downregulation of major histocompatibility complex class I and II molecules on antigen-presenting cells (Zhong et al., 1999; Ibana et al., 2011; Del Balzo et al., 2021; Wang et al., 2024). In genital tract infections, IFN-γ induces indoleamine 2,3-dioxygenase (IDO), which catabolises host tryptophan pools to restrict chlamydial growth; genital strains can evade this by utilising exogenous indole supplied by the vaginal microbiome to synthesise tryptophan (Wood et al., 2003; Ziklo et al., 2016; Wang et al., 2022). This evasion mechanism is unlikely to operate in the ocular environment, as ocular serovars harbour a non-functional *trp*BA operon and thus cannot convert indole to tryptophan (Caldwell et al., 2003; Ghasemian et al., 2025). Moreover, the ocular surface microbiome is a low-biomass community that is unlikely to provide a substantial source of exogenous indole (Scott et al., 2021). Furthermore, CtB’s selective activation of cell death pathways, including apoptosis (hsa04210) and pyroptosis (GO:0070269), alongside adaptive immune response pathways such as antigen processing and presentation (hsa04612) and JAK-STAT signalling (hsa04630), indicate an intense inflammatory infection course that likely contributes to the tissue damage in trachoma (Coers et al., 2011; Ziklo et al., 2019; Virok et al., 2021; Wang et al., 2022).

In THP-1 macrophages, CtA and CtB infection induced substantial overlap in both genes and pathways, clustering predominantly into immune and inflammatory responses, pathogen-host interactions, and tissue remodelling processes. However, despite this shared core response, CtA consistently elicited more pronounced transcriptional activation than CtB at both time points. At 4 hpi, both strains induced early activation of DNA damage response pathways, with CtA specifically upregulating DNA replication machinery and damage checkpoint signalling, whilst CtB preferentially activated DNA mismatch repair pathways. These distinct pathway signatures suggest differential strategies for managing Ct-induced genomic stress (Hayward et al., 2019). Recent findings have demonstrated that Ct disrupts homologous recombination repair through PP2A-mediated inactivation of ataxia telangiectasia mutated kinase (Mi et al., 2018), providing a molecular basis for the observed DNA damage responses.

By 24 hpi, CtA stimulated higher overall transcriptomic activity than CtB, uniquely activating key immune regulatory pathways including monocyte chemotaxis (GO:0002548) and NF-κB signalling (hsa04064), indicating sustained inflammatory programming. CtA’s specific activation of cellular chemotaxis and cAMP signalling pathways suggests enhanced capacity to modulate macrophage function. Three dominant CtA-induced genes identified in our analysis including CCL5, DNAJB6, and TNIP3 which play crucial roles in immune regulation: CCL5 mediates protective Th1 immunity through the CCR5-CCL5 axis, whilst TNIP3 functions as a negative regulator of NF-κB signalling (Sakthivel et al., 2008; Düwel et al., 2009; Shembade et al., 2010). Notably, enhanced CCL5 expression has been observed in conjunctival swabs from Gambian children with active trachoma compared to healthy controls (Natividad et al., 2010). The differential activation of NF-κB signalling pathways between CtA and CtB aligns with previous studies demonstrating functionally distinct cell line dependent NF-κB regulatory strategies amongst *Chlamydia* species (Molestina et al., 2000; Lad et al., 2007), and may represent a key mechanism underlying the varied clinical outcomes associated with different Ct genovars (Geisler et al., 2001; Xiao et al., 2005; Faris et al., 2019). The progressive reduction in THP-1 responsiveness observed from 4 to 24 hpi likely reflects temporal modulation corresponding to the chlamydial developmental cycle: robust immune activation during EB to RB conversion, followed by immune resolution during RB replication phases (Ingalls et al., 1995; Rank et al., 2010). This temporal attenuation could be mediated by sophisticated bacterial immune evasion strategies (Shaw et al., 2000), including the deubiquitinating enzyme ChlaDub1, which inhibits IκBα degradation and blocks NF-κB nuclear translocation (Le Negrate et al., 2008). Whilst CPAF was initially proposed to directly cleave the p65/RelA transcription factor, generating specific degradation products that would impair NF-κB-dependent gene transcription and reduce IL-1β-dependent IL-8 secretion (Buchholz and Stephens, 2006; Christian et al., 2010), subsequent studies have questioned whether this cleavage occurs in intact infected cells (Chen et al., 2012).

Prior transcriptomic studies of urogenital Ct strains provide useful context for interpreting our findings. CtE infection of HEp-2 epithelial cells induces early transcriptional reprogramming from 3 hpi involving metallothionein function, innate immune signalling, cytoskeletal reorganisation, and cellular stress responses, with peak dysregulation during the mid-cycle phase encompassing antigen processing and presentation, T cell receptor, mTOR, and Rap1 signalling pathways (Hayward et al., 2019; Dzakah et al., 2021; Hayward et al., 2021). Zadora et al. (2019), using an integrated phosphoproteomic and transcriptomic approach in endocervical epithelial cells infected with genovar L2, identified ERK-mediated EMT as a prominent host response alongside TNF-α/NF-κB, IL-6/JAK-STAT3, and KRAS signalling, with the ETS1/ERF-driven EMT programme associated with increased cellular invasiveness and epithelial disruption relevant to fibrotic scarring in both genital and ocular disease. Comparing these findings with our ocular genovar data, NF-κB, TNF, and cytokine-cytokine receptor interaction pathway activation appears conserved across genovars and tissue contexts; however, the pronounced ISG signature observed with CtB at 24 hpi, including IFIT1-3, MX1, MX2, OAS1/2, CXCL10, and STAT1, appears more prominent than responses reported for CtE at equivalent time points (Hayward et al., 2021; Dzakah et al., 2022), suggesting genovar-specific differences in type I IFN pathway activation. Whilst EMT was a dominant hallmark of genital L2 infection (Zadora et al., 2019), we did not observe prominent EMT enrichment in HCjE cells, potentially reflecting differences in tissue tropism, cell line characteristics, or the earlier time points examined relative to the 32–48 hpi window in which EMT signatures predominated in genital tract models. These comparisons should be interpreted cautiously given differences in cell lines.

This study has several limitations. First, whilst PCA effectively captured sources of variation, the relatively modest variance explained by PC1 and PC2 suggests that additional biological or technical factors may contribute to the observed gene expression patterns. Second, we examined only two discrete time points (4 and 24 hpi), representing the early and mid-phases of the chlamydial developmental cycle, respectively. Whilst these time points align with established transcriptomic frameworks (Hayward et al., 2019; Hayward et al., 2021) and revealed biologically meaningful strain-specific responses, the absence of a late-stage time point (≥40 hpi) precludes characterisation of host responses during RB-to-EB re-differentiation and remains a priority for future investigation. Third, the functional interpretation relied exclusively on computational enrichment analysis of GO and KEGG pathways, which, whilst informative, may not capture all relevant biological processes or reflect actual protein-level changes. Finally, whilst this study identified distinct pathway activation patterns between CtA and CtB, it did not investigate the underlying molecular mechanisms that drive these genovar-specific host responses.

To partially address the limitation of relying solely on transcriptomic data, protein-level concentrations of four key mediators; IL-6, CXCL10, CCL5, and TNF-α, were measured by multiplex immunoassay. The directional changes observed at the protein level were broadly consistent with the transcriptomic findings, supporting the biological relevance of the gene expression data. Nonetheless, our data may allow a number of mechanistic hypotheses; (*i*) the differences in ISG activation between CtB and CtA in HCjE cells, operating through STING-mediated recognition of chlamydial cyclic di-AMP, suggests that the Ct effector proteins responsible for suppressing this pathway may vary in activity or expression between CtA and CtB, (*ii*) the divergent NF-κB signalling patterns, may present genovar- and cell line- specific differences in the effectors known to modulate IκB degradation, and (*iii*) CtB’s selective activation of apoptotic and pyroptotic pathways in HCjE cells points to differential activity of effectors that inhibit host cell death during chlamydial replication. These hypotheses are testable through comparative genomic analysis of effector gene sequences between A/2497 and B/Tunis864, followed by isogenic mutant or complemented strain experiments in the cell models used here.

Given that the conjunctival epithelium represents the primary target of ocular Ct infection, the abundance of genovar-specific DEGs in epithelial cells may suggest that these cells serve as the principal site where distinct host responses to different Ct genovars are established. Our findings suggest that cell type determines fundamental response architecture whilst genovar specificity may fine-tune pathogenic outcomes. The progressive pathway enrichment in epithelial cells reflects mounting host immune and inflammatory responses to infection, alongside bacterial hijacking of cellular metabolic machinery to support replication, whereas the peak-then-decline response pattern observed in macrophages reflects the transition from an early strong response to a progressively subverted response during intracellular replication of Ct. Whilst the replication-dependent ISG cascade induced by CtB in HCjE cells might be, in principle, interruptible by early antibiotic intervention, the predominantly PAMP-driven macrophage responses at 4 hpi and the persistence of pro-fibrotic conjunctival transcriptional programmes despite doxycycline treatment reported by Derrick et al. (2019) would make it difficult to draw conclusions regarding optimal intervention timing from transcriptomic data alone. The pronounced IFN-stimulated gene signature associated with CtB infection, particularly the upregulation of CXCL10, CXCL11, and STAT1, may offer potential biomarkers for monitoring disease severity and inflammatory responses that correlate with scarring progression in trachoma. The robust inflammatory signatures induced by CtB in HCjE cells provide molecular support for clinical observations linking CtB with more severe trachomatous disease, thereby bridging these findings with epidemiological data. Collectively, these findings could support genovar-informed interventions, particularly in areas where mass drug administration may have been less effective and an identification of the circulating Ct genovars may be of benefit to control programmes.

## Data Availability

The datasets presented in this study can be found in online repositories. The names of the repository/repositories and accession number(s) can be found in the article/**Supplementary Material**.
