# Supplementary material for: Comparative transcriptomic profiling of human conjunctival epithelial cells and macrophages in response to Chlamydia trachomatis genovars A and B in early- and mid-infection cycles
Source: Front Cell Infect Microbiol. 2026 Apr 22;16:1755017. doi: 10.3389/fcimb.2026.1755017 (PMC13144089; doi:10.3389/fcimb.2026.1755017)
Supplement: Supplementary file 1 [file DataSheet1.docx]

**Supplementary Data**

**Comparative transcriptomic profiling of human conjunctival epithelial cells and macrophages in response to *Chlamydia trachomatis* genovars A and B in early- and mid-infection cycles**

Ehsan Ghasemian ^1,2 *^, Martin J. Holland ^2^

^1^Institute of Microbiology, University Hospital Center and University of Lausanne, Lausanne, Switzerland

^2^Department of Clinical Research, London School of Hygiene & Tropical Medicine, London, United Kingdom

*Correspondence

Dr Ehsan Ghasemian, Institute of Microbiology, University Hospital Center and University of Lausanne, Rue du Bugnon 48, CH-1011 Lausanne, Switzerland

ehsan.ghasemian@chuv.ch

Keywords:

*Chlamydia trachomatis*, Human Conjunctival Epithelial Cells, Macrophages, Transcriptomics, Trachoma

Data availability:

Sequencing data in the form of fastq.gz files used in this study can be accessed from the National Center for Biotechnology Information (NCBI) project accession PRJNA1133641 (Accession SAMN42382715-SAMN42382774).

**Table S1.** Read trimming statistics per sample using Cutadapt.

| Sample | Number of input reads [Millions] | Average input read length | Number of output reads [Millions] | Average output read length |
| --- | --- | --- | --- | --- |
| HCjE_CTA_4_1 | 5.62 | 101 | 5.44 | 78.43 |
| HCjE_CTA_4_2 | 3.85 | 101 | 3.67 | 73.55 |
| HCjE_CTA_4_3 | 3.85 | 101 | 3.68 | 75.1 |
| HCjE_CTA_24_1 | 6.96 | 101 | 6.7 | 79.67 |
| HCjE_CTA_24_2 | 4.64 | 101 | 4.47 | 80.26 |
| HCjE_CTA_24_3 | 4.17 | 101 | 4 | 81.75 |
| HCjE_CTA_HIA_4_1 | 5.16 | 101 | 5.01 | 82.48 |
| HCjE_CTA_HIA_4_2 | 3.35 | 101 | 3.25 | 82.92 |
| HCjE_CTA_HIA_4_3 | 1.28 | 101 | 1.16 | 68.43 |
| HCjE_CTA_HIA_24_1 | 5.27 | 101 | 5.05 | 78.84 |
| HCjE_CTA_HIA_24_2 | 4.16 | 101 | 4 | 79.1 |
| HCjE_CTA_HIA_24_3 | 5.3 | 101 | 4.99 | 77.32 |
| HCjE_CTB_4_1 | 5.14 | 101 | 4.98 | 81.68 |
| HCjE_CTB_4_2 | 4.89 | 101 | 4.73 | 80.01 |
| HCjE_CTB_4_3 | 3.27 | 101 | 3.13 | 78.46 |
| HCjE_CTB_24_1 | 4.72 | 101 | 4.56 | 78.49 |
| HCjE_CTB_24_2 | 4.86 | 101 | 4.7 | 82.64 |
| HCjE_CTB_24_3 | 4.58 | 101 | 4.4 | 76.93 |
| HCjE_CTB_HIA_4_1 | 6.11 | 101 | 5.87 | 79.79 |
| HCjE_CTB_HIA_4_2 | 3.6 | 101 | 3.48 | 79.38 |
| HCjE_CTB_HIA_4_3 | 3.96 | 101 | 3.84 | 81.08 |
| HCjE_CTB_HIA_24_1 | 4.26 | 101 | 4.13 | 78.79 |
| HCjE_CTB_HIA_24_2 | 4.2 | 101 | 4.05 | 77.19 |
| HCjE_CTB_HIA_24_3 | 6.4 | 101 | 6.19 | 80.3 |
| HCjE_Con_4_1 | 7.12 | 101 | 6.86 | 81.9 |
| HCjE_Con_4_2 | 6.45 | 101 | 6.22 | 83.17 |
| HCjE_Con_4_3 | 3.64 | 101 | 3.5 | 81.29 |
| HCjE_Con_24_1 | 4.41 | 101 | 4.27 | 77.75 |
| HCjE_Con_24_2 | 3.42 | 101 | 3.27 | 76.52 |
| HCjE_Con_24_3 | 3.47 | 101 | 3.26 | 73.13 |
| THP1_CTA_4_1 | 4.49 | 101 | 4.32 | 79.3 |
| THP1_CTA_4_2 | 3.89 | 101 | 3.77 | 81.85 |
| THP1_CTA_4_3 | 4 | 101 | 3.9 | 83.23 |
| THP1_CTA_24_1 | 5.6 | 101 | 5.39 | 81.38 |
| THP1_CTA_24_2 | 4 | 101 | 3.84 | 80.88 |
| THP1_CTA_24_3 | 3.48 | 101 | 3.26 | 74.75 |
| THP1_CTA_HIA_4_1 | 3.45 | 101 | 3.35 | 83.34 |
| THP1_CTA_HIA_4_2 | 2.96 | 101 | 2.87 | 79.41 |
| THP1_CTA_HIA_4_3 | 4.78 | 101 | 4.55 | 76.86 |
| THP1_CTA_HIA_24_1 | 3.14 | 101 | 2.98 | 75.64 |
| THP1_CTA_HIA_24_2 | 3.67 | 101 | 3.52 | 78.42 |
| THP1_CTA_HIA_24_3 | 6.17 | 101 | 5.98 | 81.02 |
| THP1_CTB_4_1 | 4.02 | 101 | 3.88 | 83.79 |
| THP1_CTB_4_2 | 4.61 | 101 | 4.43 | 78.79 |
| THP1_CTB_4_3 | 4.37 | 101 | 4.21 | 81.78 |
| THP1_CTB_24_1 | 3.47 | 101 | 3.36 | 81.65 |
| THP1_CTB_24_2 | 3.32 | 101 | 3.2 | 81.57 |
| THP1_CTB_24_3 | 4.59 | 101 | 4.4 | 79.35 |
| THP1_CTB_HIA_4_1 | 4.89 | 101 | 4.7 | 81.66 |
| THP1_CTB_HIA_4_2 | 3.99 | 101 | 3.77 | 74.57 |
| THP1_CTB_HIA_4_3 | 4.08 | 101 | 3.93 | 80.75 |
| THP1_CTB_HIA_24_1 | 5.49 | 101 | 5.2 | 75.92 |
| THP1_CTB_HIA_24_2 | 3.3 | 101 | 3.27 | 86.14 |
| THP1_CTB_HIA_24_3 | 2.93 | 101 | 2.89 | 86.32 |
| THP1_Con_4_1 | 3.32 | 101 | 3.2 | 78.84 |
| THP1_Con_4_2 | 1.5 | 101 | 1.44 | 76.73 |
| THP1_Con_4_3 | 3.95 | 101 | 3.82 | 79.53 |
| THP1_Con_24_1 | 6.2 | 101 | 5.92 | 77.71 |
| THP1_Con_24_2 | 3.06 | 101 | 2.94 | 81.42 |
| THP1_Con_24_3 | 4.11 | 101 | 3.98 | 80.57 |

**Table S2.** Read quality assessment statistics from FastQC analysis.

| Sample | Duplicates % | GC Content % | Average Sequence Length | Total Sequences | Fails % |
| --- | --- | --- | --- | --- | --- |
| HCjE_CTA_4_1_R 1 | 55.31 | 46 | 77.75 | 5437118 | 18.18 |
| HCjE_CTA_4_2_R 1 | 51.83 | 47 | 72.92 | 3666532 | 18.18 |
| HCjE_CTA_4_3_R 1 | 50.88 | 47 | 74.46 | 3678624 | 18.18 |
| HCjE_CTA_24_1_ R1 | 55.43 | 46 | 78.97 | 6695509 | 18.18 |
| HCjE_CTA_24_2_ R1 | 54.38 | 46 | 79.55 | 4472025 | 18.18 |
| HCjE_CTA_24_3_ R1 | 50.18 | 45 | 81.03 | 3995901 | 18.18 |
| HCjE_CTA_HIA_4 _1_R1 | 49.18 | 45 | 81.76 | 5010155 | 9.09 |
| HCjE_CTA_HIA_4 _2_R1 | 47.59 | 46 | 82.2 | 3249517 | 9.09 |
| HCjE_CTA_HIA_4 _3_R1 | 45.37 | 48 | 67.84 | 1163449 | 18.18 |
| HCjE_CTA_HIA_2 4_1_R1 | 54.19 | 46 | 78.14 | 5052717 | 18.18 |
| HCjE_CTA_HIA_2 4_2_R1 | 50.61 | 46 | 78.41 | 4000742 | 18.18 |
| HCjE_CTA_HIA_24_3_R1 | 48.15 | 44 | 76.63 | 4994200 | 9.09 |
| HCjE_CTB_4_1_R 1 | 48.53 | 45 | 80.96 | 4977697 | 9.09 |
| HCjE_CTB_4_2_R 1 | 53.1 | 46 | 79.31 | 4728873 | 18.18 |
| HCjE_CTB_4_3_R 1 | 49.93 | 46 | 77.77 | 3133953 | 9.09 |
| HCjE_CTB_24_1_ R1 | 53.33 | 47 | 77.82 | 4555754 | 18.18 |
| HCjE_CTB_24_2_ R1 | 48.85 | 44 | 81.92 | 4698958 | 9.09 |
| HCjE_CTB_24_3_ R1 | 53.09 | 48 | 76.26 | 4396168 | 27.27 |
| HCjE_CTB_HIA_4 _1_R1 | 53.73 | 46 | 79.08 | 5865167 | 18.18 |
| HCjE_CTB_HIA_4 _2_R1 | 50.18 | 46 | 78.69 | 3475796 | 18.18 |
| HCjE_CTB_HIA_4 _3_R1 | 49.12 | 46 | 80.37 | 3837505 | 9.09 |
| HCjE_CTB_HIA_2 4_1_R1 | 51.42 | 46 | 78.11 | 4131317 | 18.18 |
| HCjE_CTB_HIA_2 4_2_R1 | 55.73 | 47 | 76.51 | 4047313 | 27.27 |
| HCjE_CTB_HIA_2 4_3_R1 | 56.06 | 46 | 79.59 | 6193701 | 18.18 |
| HCjE_Con_4_1_R 1 | 55.05 | 45 | 81.17 | 6862080 | 18.18 |
| HCjE_Con_4_2_R 1 | 52.34 | 44 | 82.44 | 6216293 | 18.18 |
| HCjE_Con_4_3_R 1 | 47.76 | 45 | 80.57 | 3501732 | 9.09 |
| HCjE_Con_24_1_ R1 | 52.06 | 47 | 77.08 | 4268720 | 18.18 |
| HCjE_Con_24_2_ R1 | 49.44 | 46 | 75.85 | 3268176 | 9.09 |
| HCjE_Con_24_3_ R1 | 50.8 | 48 | 72.5 | 3259502 | 27.27 |
| THP1_CTA_4_1_R 1 | 56.33 | 46 | 78.59 | 4318690 | 27.27 |
| THP1_CTA_4_2_R 1 | 58.15 | 47 | 81.12 | 3771956 | 27.27 |
| THP1_CTA_4_3_R 1 | 59.16 | 47 | 82.49 | 3895121 | 27.27 |
| THP1_CTA_24_1_ R1 | 50.52 | 45 | 80.66 | 5389217 | 18.18 |
| THP1_CTA_24_2_ R1 | 48.85 | 45 | 80.16 | 3842924 | 9.09 |
| THP1_CTA_24_3_ R1 | 52.47 | 47 | 74.09 | 3258491 | 18.18 |
| THP1_CTA_HIA_4 _1_R1 | 56.14 | 46 | 82.6 | 3354842 | 27.27 |
| THP1_CTA_HIA_4 _2_R1 | 54.89 | 46 | 78.71 | 2866114 | 27.27 |
| THP1_CTA_HIA_4 _3_R1 | 52.48 | 45 | 76.18 | 4552992 | 18.18 |
| THP1_CTA_HIA_2 4_1_R1 | 49.75 | 47 | 74.98 | 2978089 | 9.09 |
| THP1_CTA_HIA_2 4_2_R1 | 50.21 | 46 | 77.73 | 3523239 | 18.18 |
| THP1_CTA_HIA_2 4_3_R1 | 57.15 | 46 | 80.31 | 5979177 | 18.18 |
| THP1_CTB_4_1_R 1 | 48.49 | 45 | 83.05 | 3879594 | 9.09 |
| THP1_CTB_4_2_R 1 | 48.26 | 45 | 78.09 | 4429426 | 9.09 |
| THP1_CTB_4_3_R 1 | 49.05 | 45 | 81.05 | 4214464 | 9.09 |
| THP1_CTB_24_1_ R1 | 48.09 | 46 | 80.93 | 3359761 | 9.09 |
| THP1_CTB_24_2_ R1 | 46.56 | 45 | 80.85 | 3198384 | 9.09 |
| THP1_CTB_24_3_ R1 | 51.37 | 45 | 78.65 | 4400766 | 18.18 |
| THP1_CTB_HIA_4 _1_R1 | 50.21 | 45 | 80.93 | 4703467 | 18.18 |
| THP1_CTB_HIA_4 _2_R1 | 50.75 | 47 | 73.92 | 3771046 | 18.18 |
| THP1_CTB_HIA_4 _3_R1 | 48.61 | 45 | 80.04 | 3933650 | 9.09 |
| THP1_CTB_HIA_2 4_1_R1 | 49.79 | 44 | 75.25 | 5203742 | 9.09 |
| THP1_CTB_HIA_2 4_2_R1 | 50.74 | 46 | 85.39 | 3266180 | 18.18 |
| THP1_CTB_HIA_2 4_3_R1 | 49.04 | 46 | 85.56 | 2889971 | 9.09 |
| THP1_Con_4_1_R 1 | 53.84 | 47 | 78.14 | 3202841 | 36.36 |
| THP1_Con_4_2_R 1 | 53.74 | 48 | 76.05 | 1443922 | 36.36 |
| THP1_Con_4_3_R 1 | 55.92 | 47 | 78.82 | 3817368 | 27.27 |
| THP1_Con_24_1_ R1 | 50.38 | 44 | 77.02 | 5924676 | 18.18 |
| THP1_Con_24_2_ R1 | 54.75 | 46 | 80.7 | 2944487 | 27.27 |
| THP1_Con_24_3_ R1 | 59.62 | 47 | 79.85 | 3978514 | 27.27 |

**Table S3.** Read alignment statistics from STAR mapping analysis.

| Sample | Number of input reads | Uniquely mapped reads number | Uniquely mapped reads % | Average input read length |
| --- | --- | --- | --- | --- |
| HCjE_CTA_4_1 | 5437118 | 3459573 | 63.63 | 78 |
| HCjE_CTA_4_2 | 3666532 | 2342327 | 63.88 | 73 |
| HCjE_CTA_4_3 | 3678624 | 2355420 | 64.03 | 75 |
| HCjE_CTA_24_1 | 6695509 | 4334216 | 64.73 | 79 |
| HCjE_CTA_24_2 | 4472025 | 2907787 | 65.02 | 80 |
| HCjE_CTA_24_3 | 3995901 | 2727267 | 68.25 | 81 |
| HCjE_CTA_HIA_4 _1 | 5010155 | 3578217 | 71.42 | 82 |
| HCjE_CTA_HIA_4 _2 | 3249517 | 2244697 | 69.08 | 82 |
| HCjE_CTA_HIA_4 _3 | 1163449 | 732595 | 62.97 | 68 |
| HCjE_CTA_HIA_2 4_1 | 5052717 | 3358681 | 66.47 | 78 |
| HCjE_CTA_HIA_2 4_2 | 4000742 | 2663156 | 66.57 | 79 |
| HCjE_CTA_HIA_2 4_3 | 4994200 | 3446040 | 69 | 77 |
| HCjE_CTB_4_1 | 4977697 | 3495059 | 70.21 | 81 |
| HCjE_CTB_4_2 | 4728873 | 3041336 | 64.31 | 80 |
| HCjE_CTB_4_3 | 3133953 | 2033053 | 64.87 | 78 |
| HCjE_CTB_24_1 | 4555754 | 2916144 | 64.01 | 78 |
| HCjE_CTB_24_2 | 4698958 | 3306386 | 70.36 | 82 |
| HCjE_CTB_24_3 | 4396168 | 2664342 | 60.61 | 76 |
| HCjE_CTB_HIA_4 _1 | 5865167 | 3904464 | 66.57 | 79 |
| HCjE_CTB_HIA_4 _2 | 3475796 | 2243706 | 64.55 | 79 |
| HCjE_CTB_HIA_4 _3 | 3837505 | 2600818 | 67.77 | 81 |
| HCjE_CTB_HIA_2 4_1 | 4131317 | 2639202 | 63.88 | 78 |
| HCjE_CTB_HIA_2 4_2 | 4047313 | 2291698 | 56.62 | 77 |
| HCjE_CTB_HIA_2 4_3 | 6193701 | 4017067 | 64.86 | 80 |
| HCjE_Con_4_1 | 6862080 | 4690485 | 68.35 | 81 |
| HCjE_Con_4_2 | 6216293 | 4413019 | 70.99 | 83 |
| HCjE_Con_4_3 | 3501732 | 2436901 | 69.59 | 81 |
| HCjE_Con_24_1 | 4268720 | 2756849 | 64.58 | 77 |
| HCjE_Con_24_2 | 3268176 | 2050458 | 62.74 | 76 |
| HCjE_Con_24_3 | 3259502 | 1971042 | 60.47 | 73 |
| THP1_CTA_4_1 | 4318690 | 2684838 | 62.17 | 79 |
| THP1_CTA_4_2 | 3771956 | 2331887 | 61.82 | 81 |
| THP1_CTA_4_3 | 3895121 | 2444804 | 62.77 | 83 |
| THP1_CTA_24_1 | 5389217 | 4003579 | 74.29 | 81 |
| THP1_CTA_24_2 | 3842924 | 2870687 | 74.7 | 80 |
| THP1_CTA_24_3 | 3258491 | 2208119 | 67.77 | 74 |
| THP1_CTA_HIA_ 4_1 | 3354842 | 2168926 | 64.65 | 83 |
| THP1_CTA_HIA_ 4_2 | 2866114 | 1812289 | 63.23 | 79 |
| THP1_CTA_HIA_ 4_3 | 4552992 | 3081279 | 67.68 | 76 |
| THP1_CTA_HIA_ 24_1 | 2978089 | 2092390 | 70.26 | 75 |
| THP1_CTA_HIA_ 24_2 | 3523239 | 2499126 | 70.93 | 78 |
| THP1_CTA_HIA_ 24_3 | 5979177 | 4237924 | 70.88 | 81 |
| THP1_CTB_4_1 | 3879594 | 2936903 | 75.7 | 83 |
| THP1_CTB_4_2 | 4429426 | 3333823 | 75.27 | 78 |
| THP1_CTB_4_3 | 4214464 | 3106703 | 73.72 | 81 |
| THP1_CTB_24_1 | 3359761 | 2385612 | 71.01 | 81 |
| THP1_CTB_24_2 | 3198384 | 2372941 | 74.19 | 81 |
| THP1_CTB_24_3 | 4400766 | 3137682 | 71.3 | 79 |
| THP1_CTB_HIA_4 _1 | 4703467 | 3520298 | 74.84 | 81 |
| THP1_CTB_HIA_4 _2 | 3771046 | 2567110 | 68.07 | 74 |
| THP1_CTB_HIA_4 _3 | 3933650 | 2872960 | 73.04 | 80 |
| THP1_CTB_HIA_2 4_1 | 5203742 | 3873484 | 74.44 | 75 |
| THP1_CTB_HIA_2 4_2 | 3266180 | 2387054 | 73.08 | 86 |
| THP1_CTB_HIA_2 4_3 | 2889971 | 2109283 | 72.99 | 86 |
| THP1_Con_4_1 | 3202841 | 1956446 | 61.08 | 78 |
| THP1_Con_4_2 | 1443922 | 831328 | 57.57 | 76 |
| THP1_Con_4_3 | 3817368 | 2385711 | 62.5 | 79 |
| THP1_Con_24_1 | 5924676 | 4330419 | 73.09 | 77 |
| THP1_Con_24_2 | 2944487 | 1955595 | 66.42 | 81 |
| THP1_Con_24_3 | 3978514 | 2449835 | 61.58 | 80 |

| ID | Description | enrichment score | enrichment score | enrichment score | enrichment score |
| --- | --- | --- | --- | --- | --- |
| CtA^*^ |  |  |  |  |  |
| GO:0048661 | positive regulation of smooth muscle cell proliferation | 0.54 |  |  |  |
| GO:0045087 | innate immune response | 0.33 |  |  |  |
| hsa04061 | Viral protein interaction with cytokine and cytokine receptor | 0.65 |  |  |  |
| hsa05323 | Rheumatoid arthritis | 0.56 |  |  |  |
| hsa04060 | Cytokine-cytokine receptor interaction | 0.49 |  |  |  |
| CtB^§^ |  |  |  |  |  |
| GO:0032731 | positive regulation of interleukin-1 beta production | 0.61 |  |  |  |
| GO:0010628 | positive regulation of gene expression | 0.36 |  |  |  |
| hsa05031 | Amphetamine addiction | 0.63 |  |  |  |
| hsa05322 | Systemic lupus erythematosus | 0.63 |  |  |  |
| Core^#^ |  |  |  |  |  |
|  |  | **CtA** | **CtB** | **CtA_HIA** | **CtB_HIA** |
| GO:0140467 | integrated stress response signaling | 0.62 | 0.64 | NA | NA |
| GO:0045444 | fat cell differentiation | 0.45 | 0.50 | NA | NA |
| GO:0006954 | inflammatory response | 0.39 | 0.43 | NA | NA |
| hsa05202 | Transcriptional misregulation in cancer | 0.476 | 0.488 | NA | NA |
| HIA^†^ |  |  |  |  |  |
|  |  |  |  | **CtA_HIA** | **CtB_HIA** |
| GO:1902895 | positive regulation of miRNA transcription |  |  | NA | 0.64 |
| GO:0006334 | nucleosome assembly |  |  | NA | 0.59 |
| GO:0000122 | negative regulation of transcription by RNA polymerase II |  |  | NA | 0.38 |
| hsa05416 | Viral myocarditis |  |  | -0.61 | NA |

**Table S4.** Pathways identified by Gene Set Enrichment Analysis (GO BP and KEGG) for HCjE cells inoculated with Ct strains A/2497 and B/Tunis864 at 4 hpi.

* Pathways unique to live CtA or shared amongst live and HIA CtA

§ Pathways unique to live CtB or shared amongst live and HIA CtB

# Pathways shared amongst live CtB or shared between live and HIA CtB), (3) core pathways (shared amongst live CtA and live CtB; live CtA and HIA CtB; live CtA, and HIA CtA and live CtB; live CtB and HIA CtA; live CtB, HIA CtB and live CtA

† Pathways unique to HIA CtA/CtB, or shared between HIA CtA/CtB

**Table S5.** Pathways identified by Gene Set Enrichment Analysis (GO BP and KEGG) for HCjE cells inoculated with Ct strains A/2497 and B/Tunis864 at 24 hpi.

| ID | Description | enrichment score | enrichment score | enrichment score | enrichment score |
| --- | --- | --- | --- | --- | --- |
| CtA^*^ |  |  |  |  |  |
| GO:0032722 | positive regulation of chemokine production | 0.57 |  |  |  |
| GO:0045333 | cellular respiration | 0.46 |  |  |  |
| GO:0030199 | collagen fibril organisation | -0.62 |  |  |  |
| hsa03060 | Protein export | 0.62 |  |  |  |
| hsa03010 | Ribosome | 0.52 |  |  |  |
| hsa00190 | Oxidative phosphorylation | 0.50 |  |  |  |
| hsa04932 | Non-alcoholic fatty liver disease | 0.47 |  |  |  |
| hsa04714 | Thermogenesis | 0.40 |  |  |  |
| hsa05020 | Prion disease | 0.38 |  |  |  |
| hsa05012 | Parkinson disease | 0.37 |  |  |  |
| hsa05010 | Alzheimer disease | 0.35 |  |  |  |
| hsa01100 | Metabolic pathways | 0.30 |  |  |  |
| hsa04820 | Cytoskeleton in muscle cells | -0.35 |  |  |  |
| hsa04974 | Protein digestion and absorption | -0.53 |  |  |  |
| CtB^§^ |  |  |  |  |  |
| GO:0045071 | negative regulation of viral genome replication | 0.80 |  |  |  |
| GO:0070269 | Pyroptosis | 0.75 |  |  |  |
| GO:0140374 | antiviral innate immune response | 0.71 |  |  |  |
| GO:0050829 | defense response to Gram-negative bacterium | 0.70 |  |  |  |
| GO:0034341 | response to type II interferon | 0.68 |  |  |  |
| GO:0071346 | cellular response to type II interferon | 0.67 |  |  |  |
| GO:0030890 | positive regulation of B cell proliferation | 0.67 |  |  |  |
| GO:0006968 | cellular defense response | 0.66 |  |  |  |
| GO:0032757 | positive regulation of interleukin-8 production | 0.66 |  |  |  |
| GO:0030593 | neutrophil chemotaxis | 0.66 |  |  |  |
| GO:0032731 | positive regulation of interleukin-1 beta production | 0.65 |  |  |  |
| GO:0071347 | cellular response to interleukin-1 | 0.64 |  |  |  |
| GO:0034142 | toll-like receptor 4 signaling pathway | 0.64 |  |  |  |
| GO:0009615 | response to virus | 0.63 |  |  |  |
| GO:0050729 | positive regulation of inflammatory response | 0.61 |  |  |  |
| GO:0071260 | cellular response to mechanical stimulus | 0.61 |  |  |  |
| GO:0098586 | cellular response to virus | 0.60 |  |  |  |
| GO:0071222 | cellular response to lipopolysaccharide | 0.59 |  |  |  |
| GO:0050830 | defense response to Gram-positive bacterium | 0.59 |  |  |  |
| GO:0042742 | defense response to bacterium | 0.59 |  |  |  |
| GO:0050731 | positive regulation of peptidyl-tyrosine phosphorylation | 0.57 |  |  |  |
| GO:0032760 | positive regulation of tumor necrosis factor production | 0.57 |  |  |  |
| GO:0032496 | response to lipopolysaccharide | 0.57 |  |  |  |
| GO:0042102 | positive regulation of T cell proliferation | 0.56 |  |  |  |
| GO:0045087 | innate immune response | 0.53 |  |  |  |
| GO:0033209 | tumor necrosis factor-mediated signaling pathway | 0.53 |  |  |  |
| GO:0002250 | adaptive immune response | 0.53 |  |  |  |
| GO:0045089 | positive regulation of innate immune response | 0.52 |  |  |  |
| GO:0048661 | positive regulation of smooth muscle cell proliferation | 0.52 |  |  |  |
| GO:0001819 | positive regulation of cytokine production | 0.52 |  |  |  |
| GO:0051092 | positive regulation of NF-kappaB transcription factor activity | 0.51 |  |  |  |
| GO:0070374 | positive regulation of ERK1 and ERK2 cascade | 0.50 |  |  |  |
| GO:0043123 | positive regulation of canonical NF-kappaB signal transduction | 0.49 |  |  |  |
| GO:0051897 | positive regulation of phosphatidylinositol 3-kinase/protein kinase B signal transduction | 0.46 |  |  |  |
| GO:0008285 | negative regulation of cell population proliferation | 0.35 |  |  |  |
| hsa04672 | Intestinal immune network for IgA production | 0.77 |  |  |  |
| hsa04940 | Type I diabetes mellitus | 0.74 |  |  |  |
| hsa05143 | African trypanosomiasis | 0.67 |  |  |  |
| hsa04623 | Cytosolic DNA-sensing pathway | 0.67 |  |  |  |
| hsa04622 | RIG-I-like receptor signaling pathway | 0.63 |  |  |  |
| hsa05144 | Malaria | 0.62 |  |  |  |
| hsa05133 | Pertussis | 0.58 |  |  |  |
| hsa04612 | Antigen processing and presentation | 0.58 |  |  |  |
| hsa04064 | NF-kappa B signaling pathway | 0.57 |  |  |  |
| hsa04640 | Hematopoietic cell lineage | 0.57 |  |  |  |
| hsa05416 | Viral myocarditis | 0.57 |  |  |  |
| hsa05140 | Leishmaniasis | 0.55 |  |  |  |
| hsa03250 | Viral life cycle - HIV-1 | 0.55 |  |  |  |
| hsa05160 | Hepatitis C | 0.54 |  |  |  |
| hsa05152 | Tuberculosis | 0.47 |  |  |  |
| hsa04210 | Apoptosis | 0.47 |  |  |  |
| hsa05169 | Epstein-Barr virus infection | 0.46 |  |  |  |
| hsa04630 | JAK-STAT signaling pathway | 0.45 |  |  |  |
| hsa04217 | Necroptosis | 0.45 |  |  |  |
| hsa05130 | Pathogenic Escherichia coli infection | 0.44 |  |  |  |
| hsa05161 | Hepatitis B | 0.43 |  |  |  |
| hsa05168 | Herpes simplex virus 1 infection | 0.37 |  |  |  |
| Core^#^ |  |  |  |  |  |
|  |  | **CtA** | **CtB** | **CtA_HIA** | **CtB_HIA** |
| GO:0070098 | chemokine-mediated signaling pathway | 0.73 | 0.80 | 0.76 | NA |
| GO:0051607 | defense response to virus | 0.38 | 0.67 | NA | NA |
| GO:0019221 | cytokine-mediated signaling pathway | NA | 0.59 | 0.46 | 0.40 |
| GO:0035914 | skeletal muscle cell differentiation | 0.58 | 0.56 | 0.61 | NA |
| GO:0071356 | cellular response to tumor necrosis factor | 0.45 | 0.54 | 0.51 | NA |
| GO:0006954 | inflammatory response | NA | 0.49 | 0.37 | NA |
| GO:0006935 | chemotaxis | NA | 0.47 | 0.49 | NA |
| GO:0008284 | positive regulation of cell population proliferation | 0.32 | NA | NA | 0.33 |
| hsa04061 | Viral protein interaction with cytokine and cytokine receptor | 0.61 | 0.74 | 0.73 | 0.66 |
| hsa05323 | Rheumatoid arthritis | 0.66 | 0.71 | 0.77 | 0.69 |
| hsa05134 | Legionellosis | 0.64 | 0.69 | 0.61 | 0.57 |
| hsa05164 | Influenza A | 0.52 | 0.66 | 0.51 | NA |
| hsa04060 | Cytokine-cytokine receptor interaction | 0.52 | 0.66 | 0.65 | 0.59 |
| hsa04657 | IL-17 signaling pathway | 0.49 | 0.64 | 0.62 | 0.58 |
| hsa04620 | Toll-like receptor signaling pathway | 0.54 | 0.64 | 0.56 | NA |
| hsa04668 | TNF signaling pathway | 0.43 | 0.61 | 0.58 | 0.48 |
| hsa04621 | NOD-like receptor signaling pathway | NA | 0.60 | 0.46 | NA |
| hsa05162 | Measles | 0.45 | 0.54 | NA | NA |
| hsa05171 | Coronavirus disease - COVID-19 | 0.45 | 0.53 | 0.45 | NA |
| hsa05142 | Chagas disease | 0.47 | 0.50 | 0.59 | 0.48 |
| hsa04062 | Chemokine signaling pathway | 0.45 | 0.50 | 0.51 | NA |
| hsa05417 | Lipid and atherosclerosis | 0.40 | 0.49 | 0.45 | NA |
| hsa05167 | Kaposi sarcoma-associated herpesvirus infection | NA | 0.47 | 0.46 | NA |
| hsa05163 | Human cytomegalovirus infection | 0.40 | 0.45 | 0.44 | NA |
| hsa05202 | Transcriptional misregulation in cancer | NA | 0.43 | 0.48 | NA |
| hsa05150 | Staphylococcus aureus infection | -0.66 | NA | NA | -0.70 |
| HIA^†^ |  |  |  |  |  |
|  |  |  |  | **CtA_HIA** | **CtB_HIA** |
| GO:0035335 | peptidyl-tyrosine dephosphorylation |  |  | 0.75 | NA |
| GO:2001240 | negative regulation of extrinsic apoptotic signaling pathway in absence of ligand |  |  | 0.73 | NA |
| GO:0014823 | response to activity |  |  | 0.58 | NA |
| GO:0070371 | ERK1 and ERK2 cascade |  |  | 0.49 | 0.41 |
| GO:0043066 | negative regulation of apoptotic process |  |  | 0.34 | NA |
| GO:0010628 | positive regulation of gene expression |  |  | 0.31 | NA |
| GO:0006270 | DNA replication initiation |  |  | NA | 0.61 |
| GO:0090630 | activation of GTPase activity |  |  | NA | -0.52 |
| hsa05321 | Inflammatory bowel disease |  |  | 0.68 | 0.65 |
| hsa05120 | Epithelial cell signaling in Helicobacter pylori infection |  |  | 0.57 | NA |
| hsa04936 | Alcoholic liver disease |  |  | 0.52 | NA |
| hsa04933 | AGE-RAGE signaling pathway in diabetic complications |  |  | 0.51 | NA |
| hsa05208 | Chemical carcinogenesis - reactive oxygen species |  |  | 0.45 | NA |
| hsa04010 | MAPK signaling pathway |  |  | 0.41 | NA |
| hsa05200 | Pathways in cancer |  |  | 0.38 | NA |
| hsa05022 | Pathways of neurodegeneration - multiple diseases |  |  | 0.37 | NA |

* Pathways unique to live CtA or shared amongst live and HIA CtA

§ Pathways unique to live CtB or shared amongst live and HIA CtB

# Pathways shared amongst live CtB or shared between live and HIA CtB), (3) core pathways (shared amongst live CtA and live CtB; live CtA and HIA CtB; live CtA, and HIA CtA and live CtB; live CtB and HIA CtA; live CtB, HIA CtB and live CtA

† Pathways unique to HIA CtA/CtB, or shared between HIA CtA/CtB

**Table S6.** Pathways identified by Gene Set Enrichment Analysis (GO BP and KEGG) for HCjE cells inoculated with Ct strains A/2497 and B/Tunis864 at 4 hpi.

| ID | Description | enrichment score | enrichment score | enrichment score | enrichment score |
| --- | --- | --- | --- | --- | --- |
| CtA^*^ |  |  |  |  |  |
| GO:1902895 | positive regulation of miRNA transcription | 0.56 |  |  |  |
| GO:0007204 | positive regulation of cytosolic calcium ion concentration | 0.53 |  |  |  |
| GO:0098609 | cell-cell adhesion | 0.40 |  |  |  |
| GO:0001525 | angiogenesis | 0.37 |  |  |  |
| GO:0007267 | cell-cell signaling | 0.32 |  |  |  |
| GO:0051056 | regulation of small GTPase mediated signal transduction | -0.35 |  |  |  |
| GO:0006260 | DNA replication | -0.36 |  |  |  |
| GO:0000077 | DNA damage checkpoint signaling | -0.39 |  |  |  |
| hsa04512 | ECM-receptor interaction | 0.57 |  |  |  |
| hsa05213 | Endometrial cancer | 0.55 |  |  |  |
| hsa04024 | cAMP signaling pathway | 0.44 |  |  |  |
| hsa05163 | Human cytomegalovirus infection | 0.41 |  |  |  |
| hsa05200 | Pathways in cancer | 0.36 |  |  |  |
| CtB^§^ |  |  |  |  |  |
| GO:0042311 | vasodilation | 0.66 |  |  |  |
| GO:0034605 | cellular response to heat | 0.54 |  |  |  |
| GO:0019221 | cytokine-mediated signaling pathway | 0.46 |  |  |  |
| GO:0045944 | positive regulation of transcription by RNA polymerase II | 0.30 |  |  |  |
| GO:0031297 | replication fork processing | -0.63 |  |  |  |
| hsa04930 | Type II diabetes mellitus | 0.67 |  |  |  |
| hsa05120 | Epithelial cell signaling in Helicobacter pylori infection | 0.56 |  |  |  |
| hsa03430 | Mismatch repair | -0.71 |  |  |  |
| Core^#^ |  |  |  |  |  |
|  |  | **CtA** | **CtB** | **CtA_HIA** | **CtB_HIA** |
| GO:0002548 | monocyte chemotaxis | NA | 0.72 | 0.69 | 0.66 |
| GO:0070098 | chemokine-mediated signaling pathway | 0.74 | 0.71 | 0.76 | 0.62 |
| GO:0042531 | positive regulation of tyrosine phosphorylation of STAT protein | 0.67 | 0.71 | 0.69 | NA |
| GO:0003180 | aortic valve morphogenesis | 0.67 | 0.69 | 0.71 | NA |
| GO:0030593 | neutrophil chemotaxis | 0.70 | 0.65 | NA | NA |
| GO:0071347 | cellular response to interleukin-1 | 0.64 | 0.62 | 0.62 | 0.58 |
| GO:0071222 | cellular response to lipopolysaccharide | 0.52 | 0.56 | 0.52 | 0.50 |
| GO:0050729 | positive regulation of inflammatory response | NA | 0.54 | 0.56 | NA |
| GO:0071356 | cellular response to tumor necrosis factor | 0.53 | 0.52 | 0.56 | 0.49 |
| GO:0007219 | Notch signaling pathway | 0.44 | 0.42 | 0.41 | NA |
| GO:0006954 | inflammatory response | 0.41 | 0.42 | 0.42 | 0.42 |
| GO:0007186 | G protein-coupled receptor signaling pathway | 0.40 | 0.41 | 0.42 | 0.43 |
| GO:0008285 | negative regulation of cell population proliferation | 0.35 | 0.41 | 0.39 | 0.38 |
| GO:0043066 | negative regulation of apoptotic process | 0.35 | 0.36 | 0.35 | 0.37 |
| GO:0007155 | cell adhesion | 0.37 | 0.35 | 0.37 | 0.37 |
| GO:0010628 | positive regulation of gene expression | 0.33 | 0.33 | 0.35 | 0.32 |
| GO:0030335 | positive regulation of cell migration | 0.40 | NA | 0.43 | 0.41 |
| GO:0000724 | double-strand break repair via homologous recombination | -0.40 | -0.45 | NA | -0.46 |
| hsa04061 | Viral protein interaction with cytokine and cytokine receptor | 0.68 | 0.69 | 0.69 | 0.64 |
| hsa04060 | Cytokine-cytokine receptor interaction | 0.63 | 0.67 | 0.62 | 0.62 |
| hsa04668 | TNF signaling pathway | 0.59 | 0.61 | 0.63 | 0.62 |
| hsa05323 | Rheumatoid arthritis | 0.64 | 0.68 | 0.63 | 0.60 |
| hsa04062 | Chemokine signaling pathway | 0.50 | 0.46 | 0.46 | NA |
| hsa05144 | Malaria | 0.71 | 0.68 | 0.69 | NA |
| hsa05145 | Toxoplasmosis | 0.52 | 0.50 | 0.51 | NA |
| hsa04620 | Toll-like receptor signaling pathway | 0.51 | 0.54 | 0.51 | NA |
| hsa04625 | C-type lectin receptor signaling pathway | 0.52 | 0.54 | 0.55 | 0.54 |
| hsa05134 | Legionellosis | NA | 0.63 | 0.59 | NA |
| hsa05146 | Amoebiasis | 0.57 | 0.57 | 0.55 | 0.55 |
| hsa04657 | IL-17 signaling pathway | 0.55 | 0.58 | 0.54 | 0.55 |
| hsa05417 | Lipid and atherosclerosis | 0.40 | 0.46 | 0.42 | NA |
| hsa04064 | NF-kappa B signaling pathway | 0.57 | 0.57 | 0.54 | 0.53 |
| hsa04630 | JAK-STAT signaling pathway | 0.53 | 0.55 | 0.50 | 0.56 |
| HIA^†^ |  |  |  |  |  |
|  |  |  |  | **CtA_HIA** | **CtB_HIA** |
| GO:0001974 | blood vessel remodeling |  |  | 0.68 | NA |
| GO:0045600 | positive regulation of fat cell differentiation |  |  | 0.63 | NA |
| GO:0043616 | keratinocyte proliferation |  |  | 0.60 | NA |
| GO:0030216 | keratinocyte differentiation |  |  | 0.58 | NA |
| GO:0001938 | positive regulation of endothelial cell proliferation |  |  | 0.55 | NA |
| GO:0014032 | neural crest cell development |  |  | 0.55 | NA |
| GO:0048661 | positive regulation of smooth muscle cell proliferation |  |  | 0.52 | NA |
| GO:0008284 | positive regulation of cell population proliferation |  |  | 0.37 | NA |

* Pathways unique to live CtA or shared amongst live and HIA CtA

§ Pathways unique to live CtB or shared amongst live and HIA CtB

# Pathways shared amongst live CtA and CtB; live CtA and HIA Ct; live CtA, and HIA CtA and CtB; live CtB and HIA CtA; live CtA, HIA CtA and CtB

† Pathways unique to HIA CtA/CtB, or shared between HIA CtA/CtB

**Table S7.** Pathways identified by Gene Set Enrichment Analysis (GO BP and KEGG) for HCjE cells inoculated with Ct strains A/2497 and B/Tunis864 at 24 hpi.

| ID | Description | enrichment score | enrichment score | enrichment score | enrichment score |
| --- | --- | --- | --- | --- | --- |
| CtA^*^ |  |  |  |  |  |
| GO:0002548 | monocyte chemotaxis | 0.78 |  |  |  |
| GO:0007186 | G protein-coupled receptor signaling pathway | 0.36 |  |  |  |
| GO:0000070 | mitotic sister chromatid segregation | -0.42 |  |  |  |
| hsa04064 | NF-kappa B signaling pathway | 0.51 |  |  |  |
| hsa05171 | Coronavirus disease - COVID-19 | 0.41 |  |  |  |
| CtB^§^ |  |  |  |  |  |
|  |  |  |  |  |  |
| Core^#^ |  |  |  |  |  |
|  |  | **CtA** | **CtB** | **CtA_HIA** | **CtB_HIA** |
| GO:0030593 | neutrophil chemotaxis | 0.71 | 0.75 | NA | 0.77 |
| GO:0070098 | chemokine-mediated signaling pathway | 0.68 | 0.73 | 0.64 | 0.74 |
| GO:0071347 | cellular response to interleukin-1 | 0.65 | 0.65 | 0.61 | 0.67 |
| GO:0071222 | cellular response to lipopolysaccharide | NA | 0.52 | 0.44 | 0.47 |
| GO:0006954 | inflammatory response | 0.40 | 0.39 | 0.34 | 0.39 |
| hsa04061 | Viral protein interaction with cytokine and cytokine receptor | 0.70 | 0.70 | 0.64 | 0.74 |
| hsa04657 | IL-17 signaling pathway | 0.63 | 0.62 | NA | 0.64 |
| hsa04060 | Cytokine-cytokine receptor interaction | 0.59 | 0.58 | 0.53 | 0.62 |
| hsa05323 | Rheumatoid arthritis | 0.59 | 0.56 | NA | 0.61 |
| hsa04668 | TNF signaling pathway | 0.50 | 0.48 | NA | 0.53 |
| hsa04062 | Chemokine signaling pathway | 0.48 | 0.54 | NA | 0.49 |
| HIA^†^ |  |  |  |  |  |
|  |  |  |  | **CtA_HIA** | **CtB_HIA** |
| GO:0046676 | negative regulation of insulin secretion |  |  | NA | 0.72 |

* Pathways unique to live CtA or shared amongst live and HIA CtA

§ Pathways unique to live CtB or shared amongst live and HIA CtB

# Pathways shared amongst live CtA and CtB; live CtA and HIA Ct; live CtA, and HIA CtA and CtB; live CtB and HIA CtA; live CtA, HIA CtA and CtB

† Pathways unique to HIA CtA/CtB, or shared between HIA CtA/CtB

**
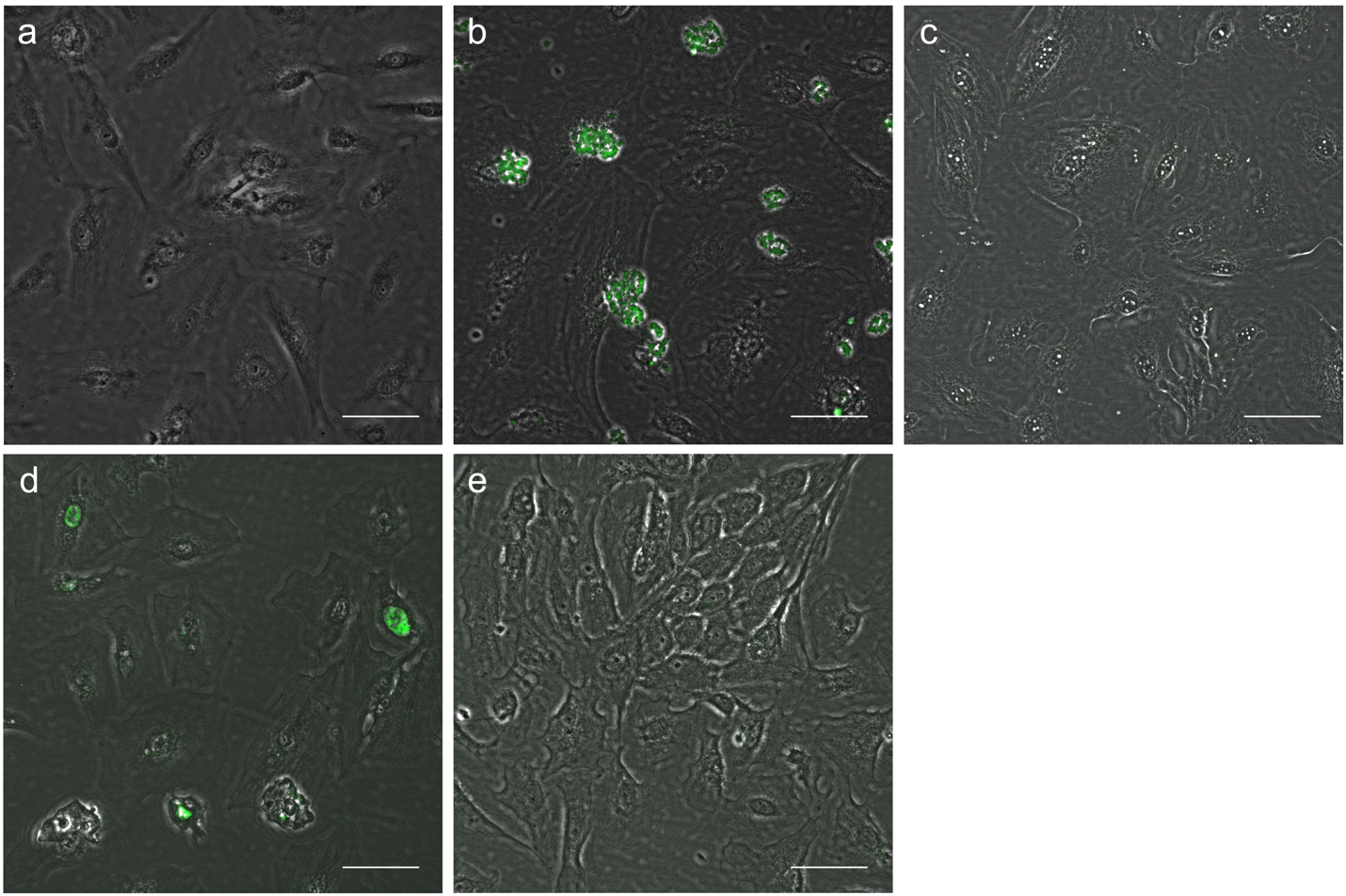
Fig. S1.** Immunofluorescence detection of CtA and CtB in HCjE cells. Ct inclusions were visualised using anti-LPS antibodies (Pathfinder Chlamydia Confirmation System). (**a**) Uninoculated control, (**b**) live CtA, (**c**) HIA CtA, (d) live CtB, (**e**) HIA CtB. Scale bars = 50 μm.

**
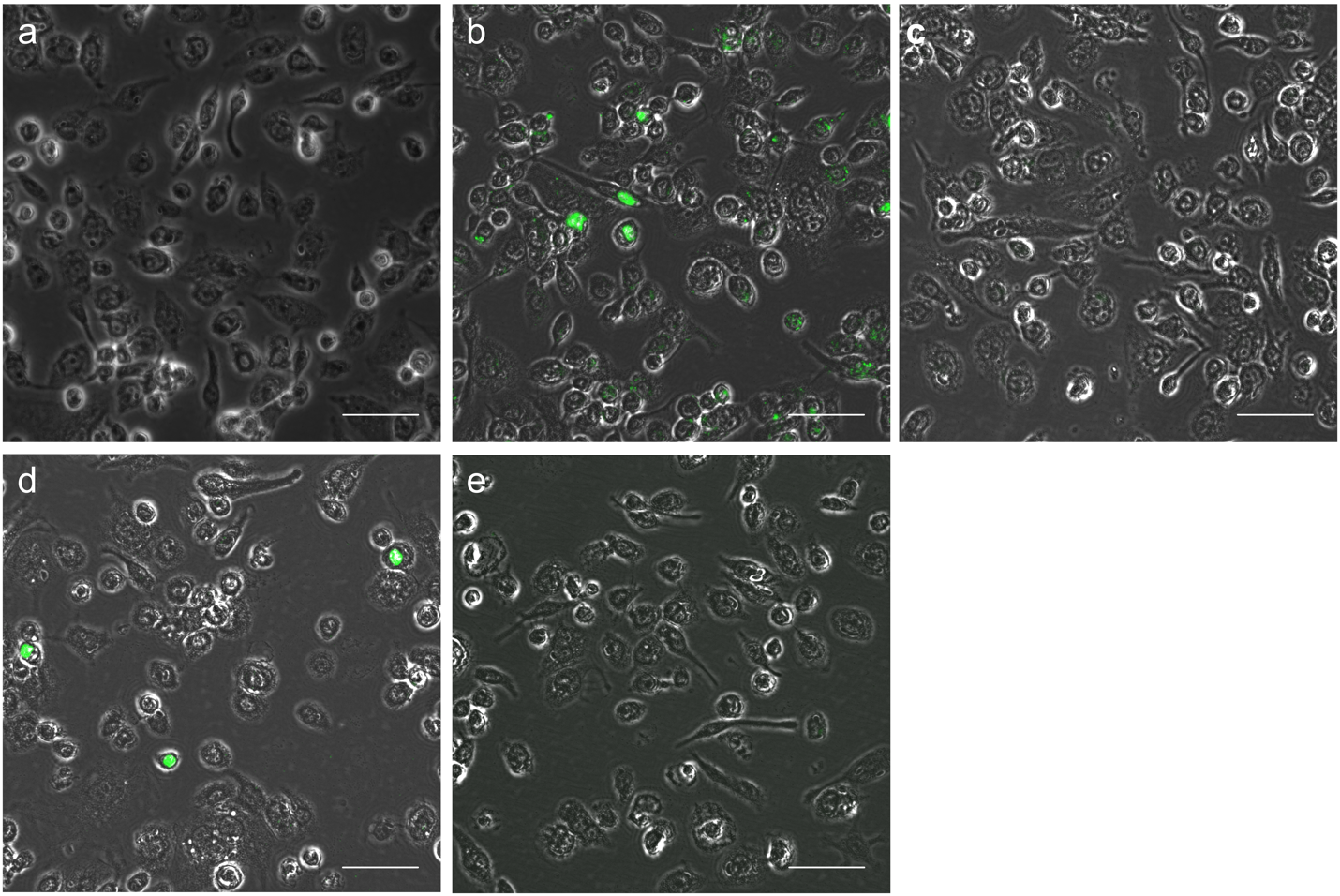
**

**Fig. S2.** Immunofluorescence detection of CtA and CtB in THP1 cells. Ct inclusions were visualised using anti-LPS antibodies (Pathfinder Chlamydia Confirmation System). (**a**) Uninoculated control, (**b**) live CtA, (**c**) HIA CtA, (d) live CtB, (**e**) HIA CtB. Scale bars = 50 μm.

**Fig. S3.** (a) Read length distribution from FastQC analysis. (b) Per-read quality score distribution from FastQC analysis.


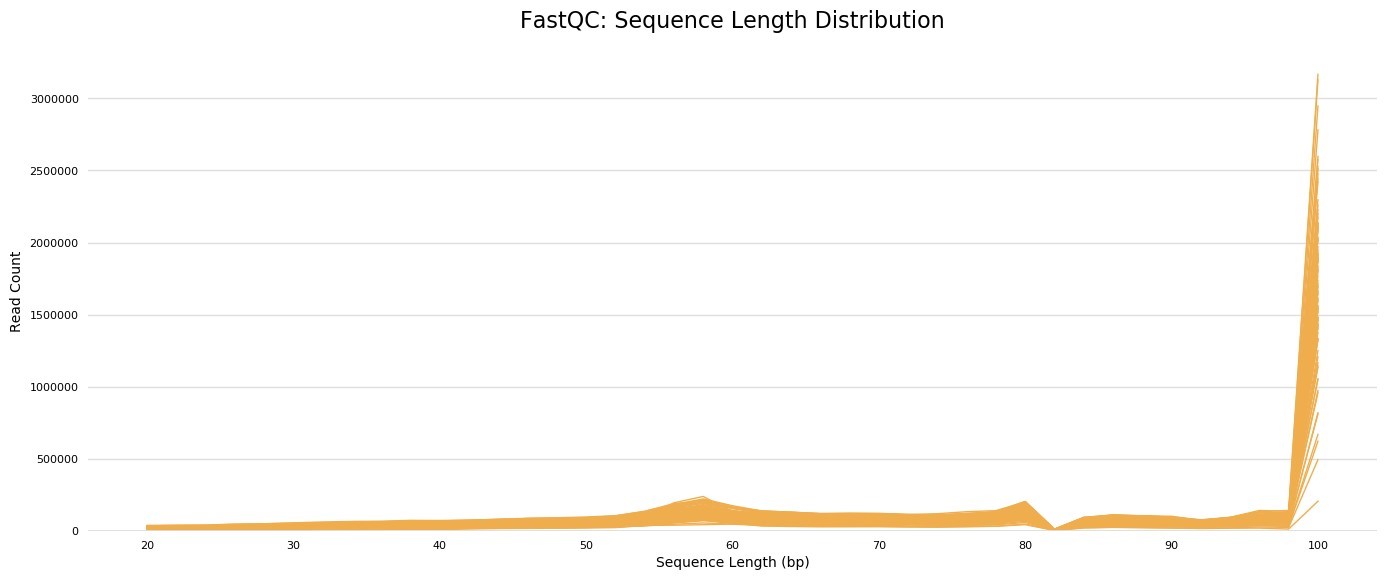

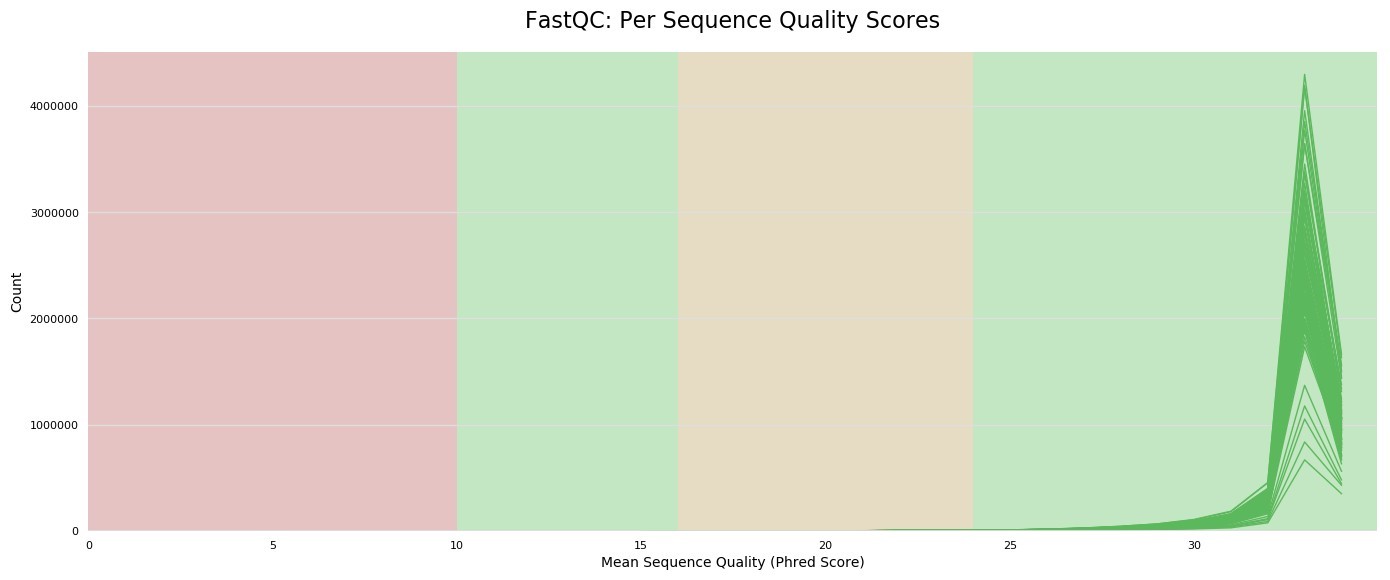


a.

b.


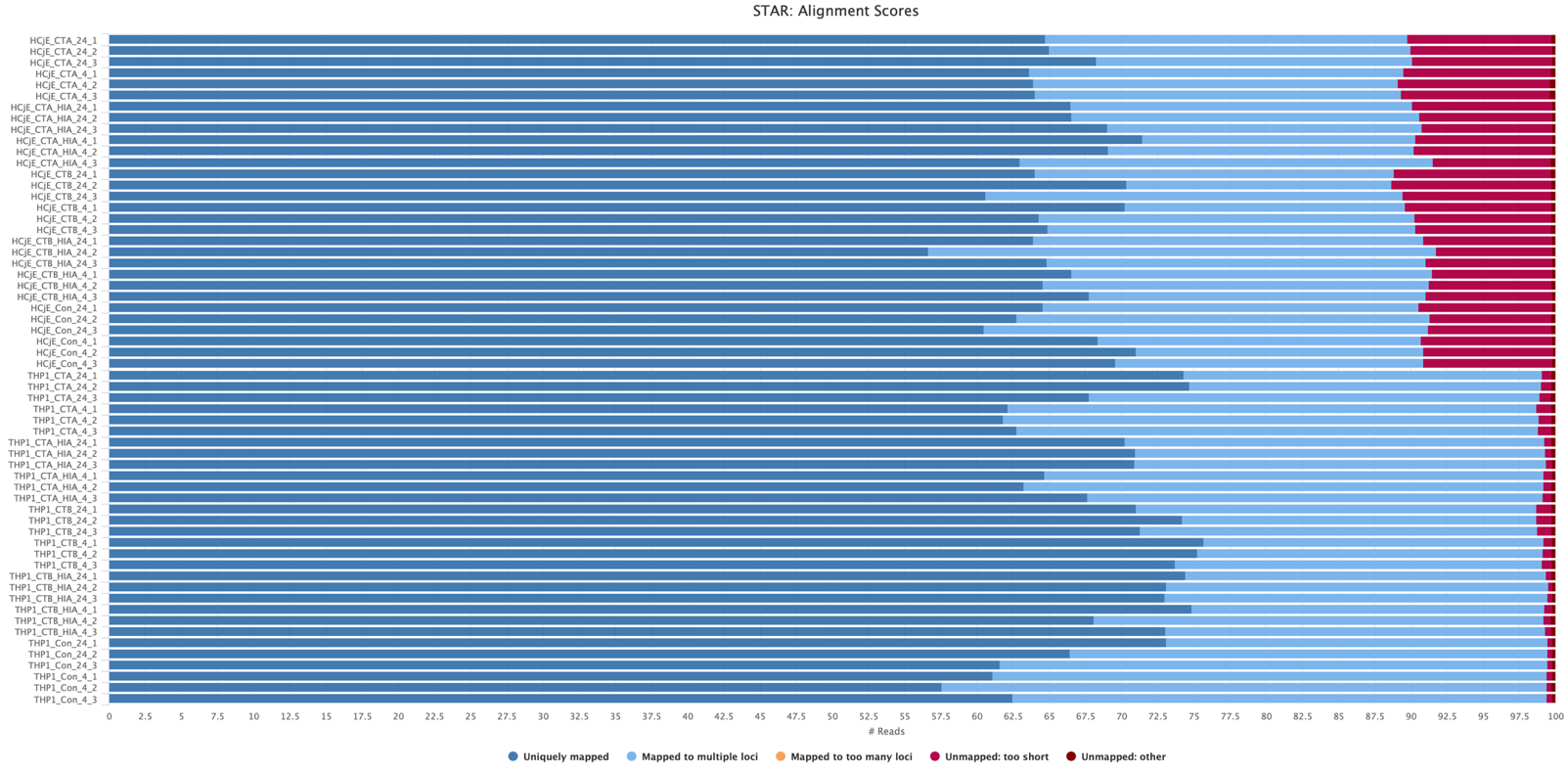
**Fig. S4.** Read alignment summary showing the proportion of reads in each alignment category per sample.

**
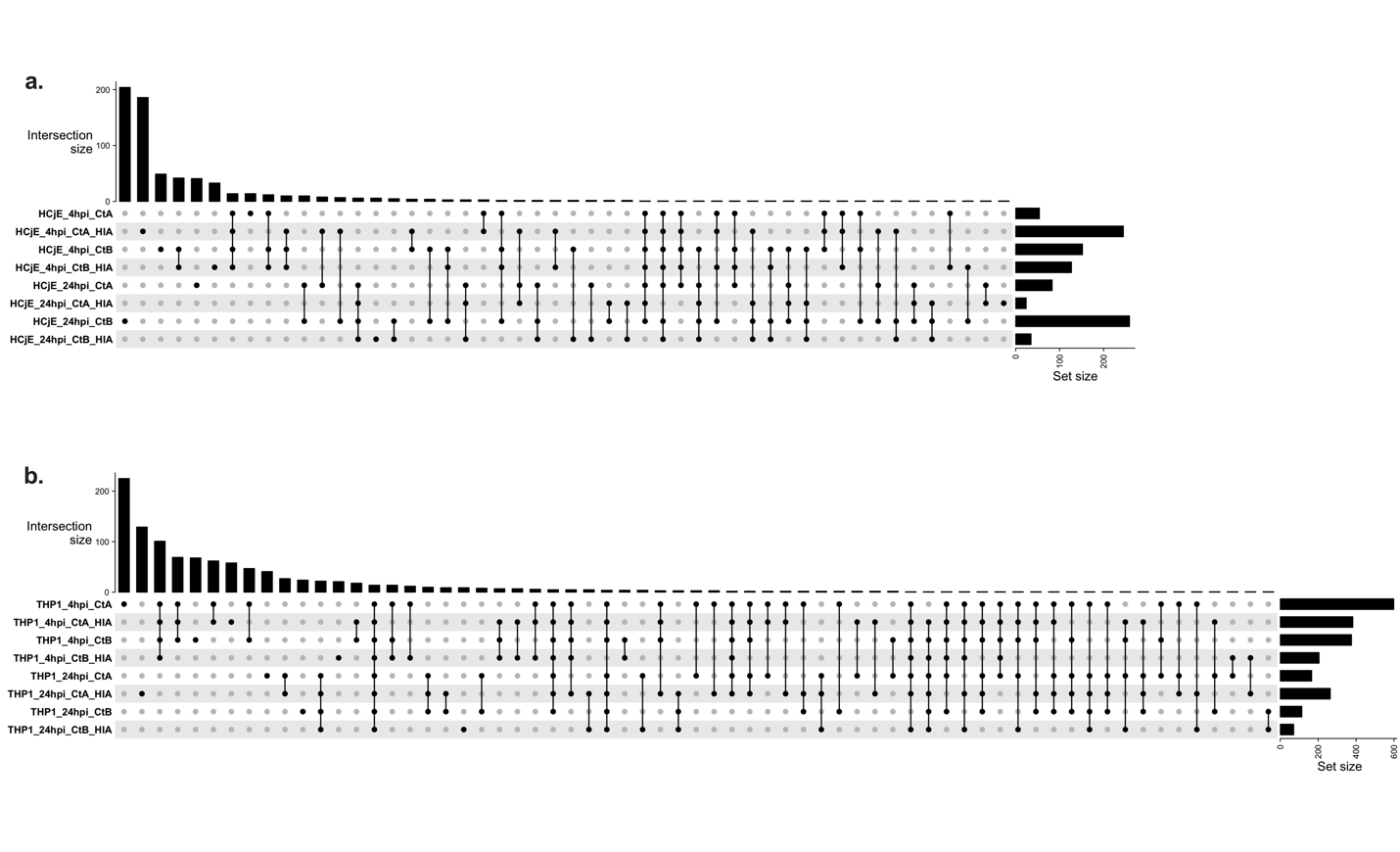
Fig. S5.** UpSet plot showing the intersection of DEGs across various inoculation conditions in HCjE (**a**) and THP1 (**b**) cells. The plot visualises the number of DEGs unique to each condition and shared among combinations of conditions. Vertical bars represent the size of each intersection set, while filled dots below indicate which conditions are involved in each intersection.

**
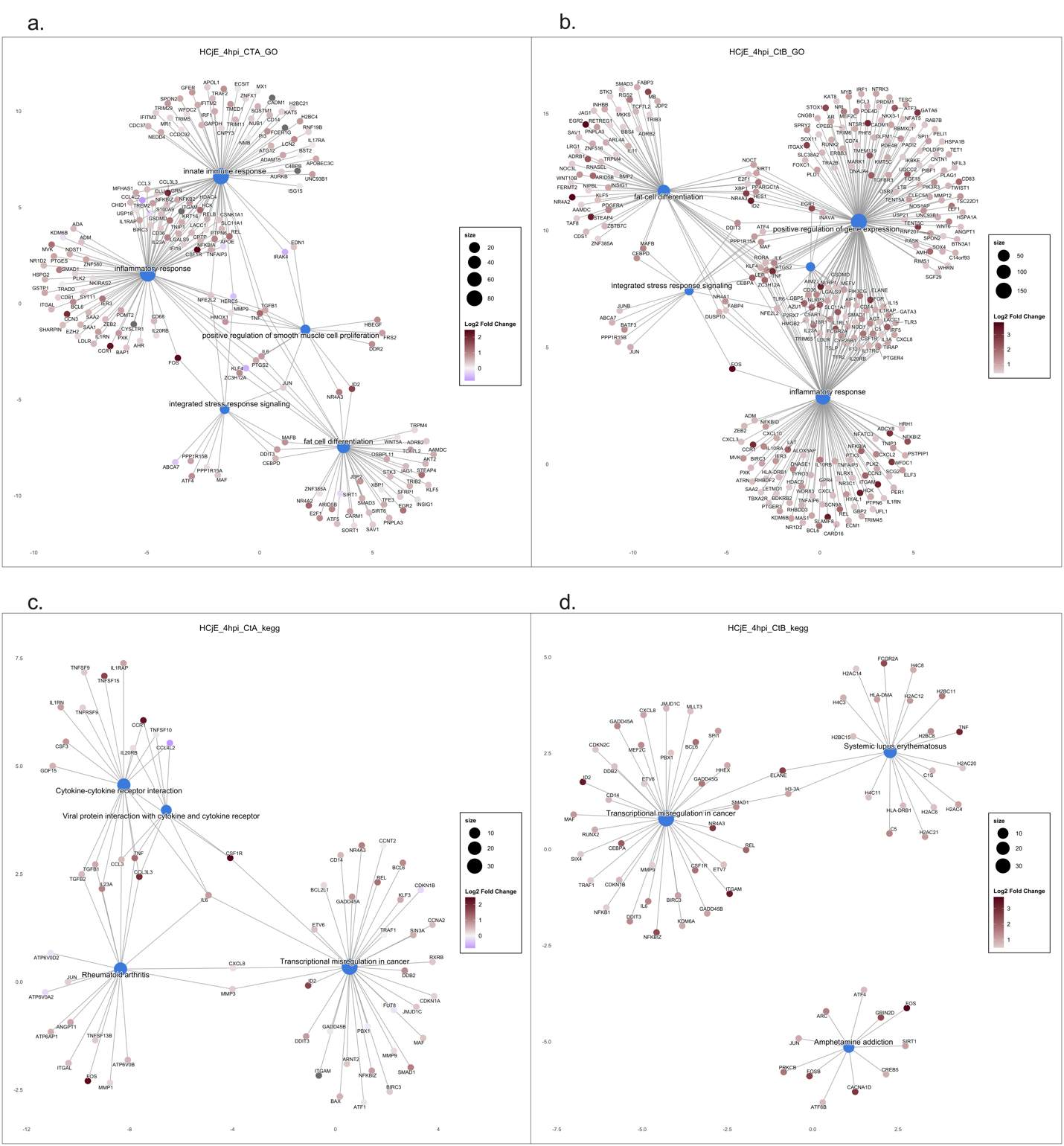
**

**Fig. S6.**

**
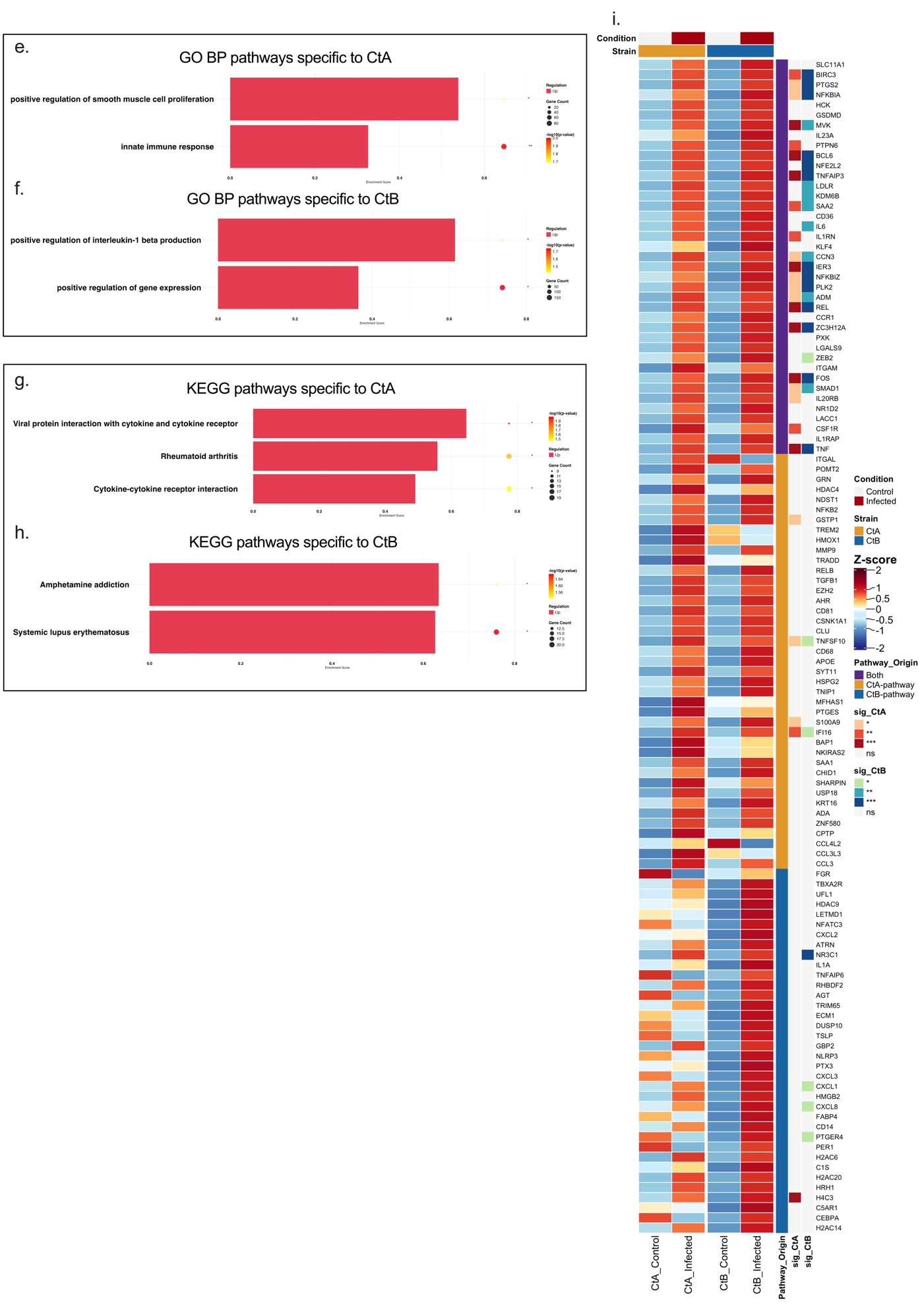
**

**Fig. S6.** Pathway enrichment analysis and differential gene expression profiles in HCjE cells infected with Ct strains A/2497 and B/Tunis864 at 4 hpi. (**a**-**d**) Pathway-gene interaction networks for significantly enriched GO BP (**a**, **b**) and KEGG (**c**, **d**) pathways following CtA and CtB infection. Pathway nodes are sized by significance; gene nodes are coloured by log2FC (blue: downregulated; red: upregulated). (**e**-**h**) Strain-specific pathway enrichment profiles for GO BP (**e**, **f**) and KEGG (**g**, **h**). Bars show enrichment magnitude/direction; overlaid circles represent gene count (size) and significance (colour intensity, -log10 adjusted *P*-value). (**i**) Heatmap of differentially expressed pathway-associated genes (adjusted P < 0.05). Columns show mean Z-scored expression for controls and infected samples (CtA: A/2497; CtB: B/Tunis864). Row annotations indicate pathway origin (red: CtA-pathway; blue: CtB-pathway; orange: both) and strain-specific significance levels. Column annotations denote condition (grey: control; orange: infected) and strain (orange: CtA; blue: CtB). Colour scale: blue (low) to red (high) expression. Significance: *** *P* < 0.001, ** *P* < 0.01, * *P* < 0.05.

**
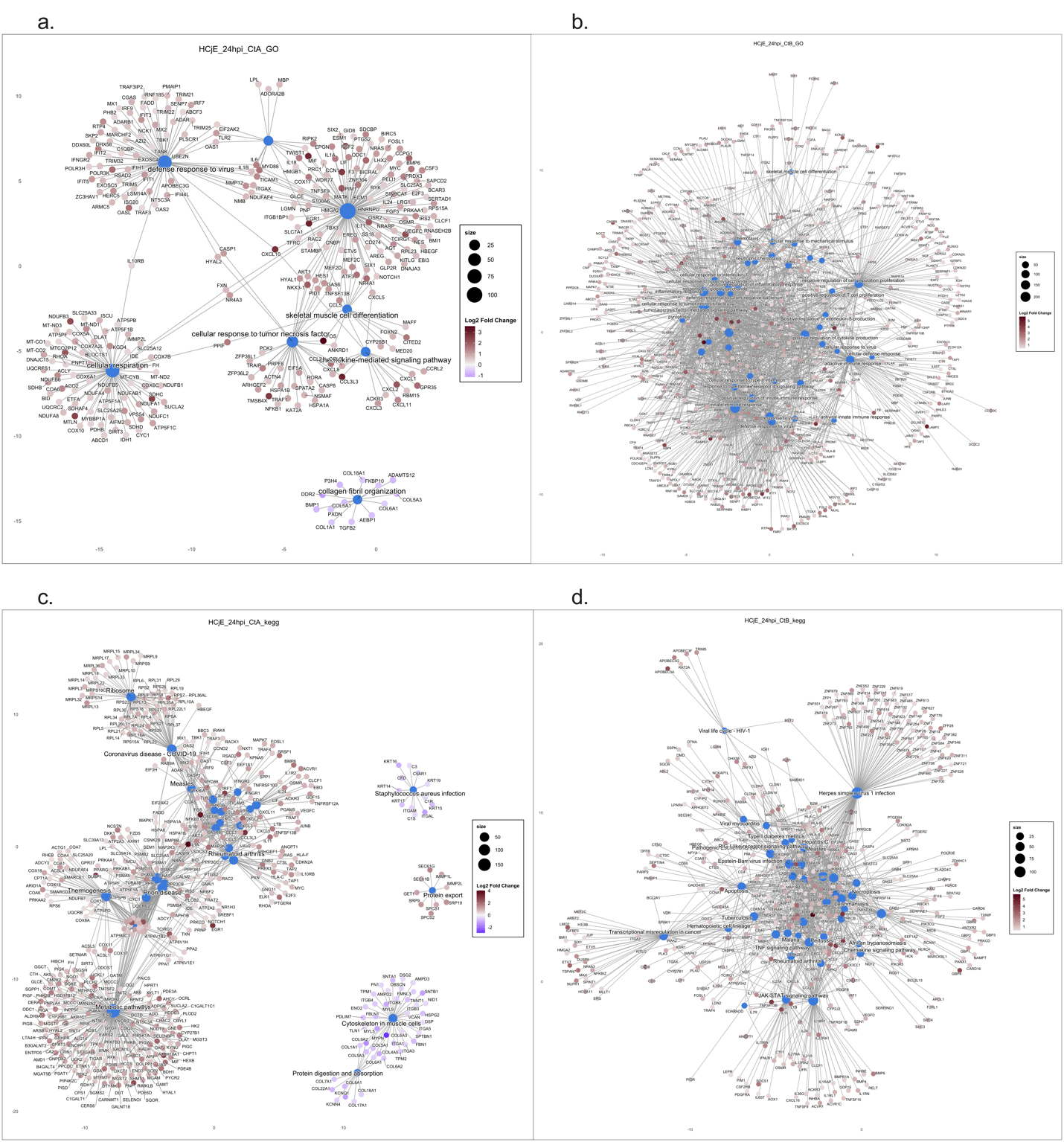
**

**Fig. S7.**

**
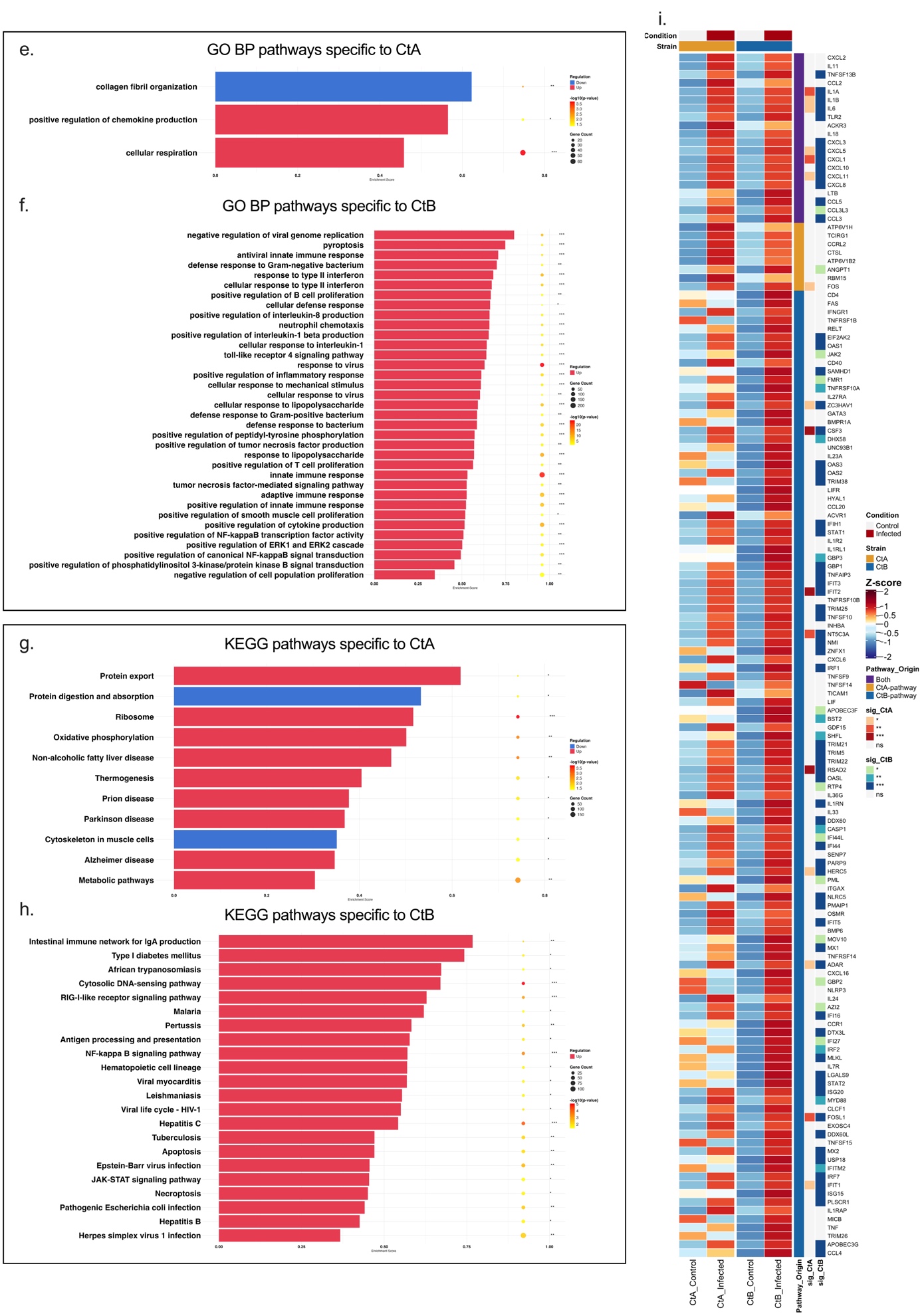
**

**Fig. S7.** Pathway enrichment analysis and differential gene expression profiles in HCjE cells infected with Ct strains A/2497 and B/Tunis864 at 24 hpi. (**a**-**d**) Pathway-gene interaction networks for significantly enriched GO BP (**a**, **b**) and KEGG (**c**, **d**) pathways following CtA and CtB infection. Pathway nodes are sized by significance; gene nodes are coloured by log2FC (blue: downregulated; red: upregulated). (**e**-**h**) Strain-specific pathway enrichment profiles for GO BP (**e**, **f**) and KEGG (**g**, **h**). Bars show enrichment magnitude/direction; overlaid circles represent gene count (size) and significance (colour intensity, -log10 adjusted *P*-value). (**i**) Heatmap of differentially expressed pathway-associated genes (adjusted P < 0.05). Columns show mean Z-scored expression for controls and infected samples (CtA: A/2497; CtB: B/Tunis864). Row annotations indicate pathway origin (red: CtA-pathway; blue: CtB-pathway; orange: both) and strain-specific significance levels. Column annotations denote condition (grey: control; orange: infected) and strain (orange: CtA; blue: CtB). Colour scale: blue (low) to red (high) expression. Significance: *** *P* < 0.001, ** *P* < 0.01, * *P* < 0.05.

**
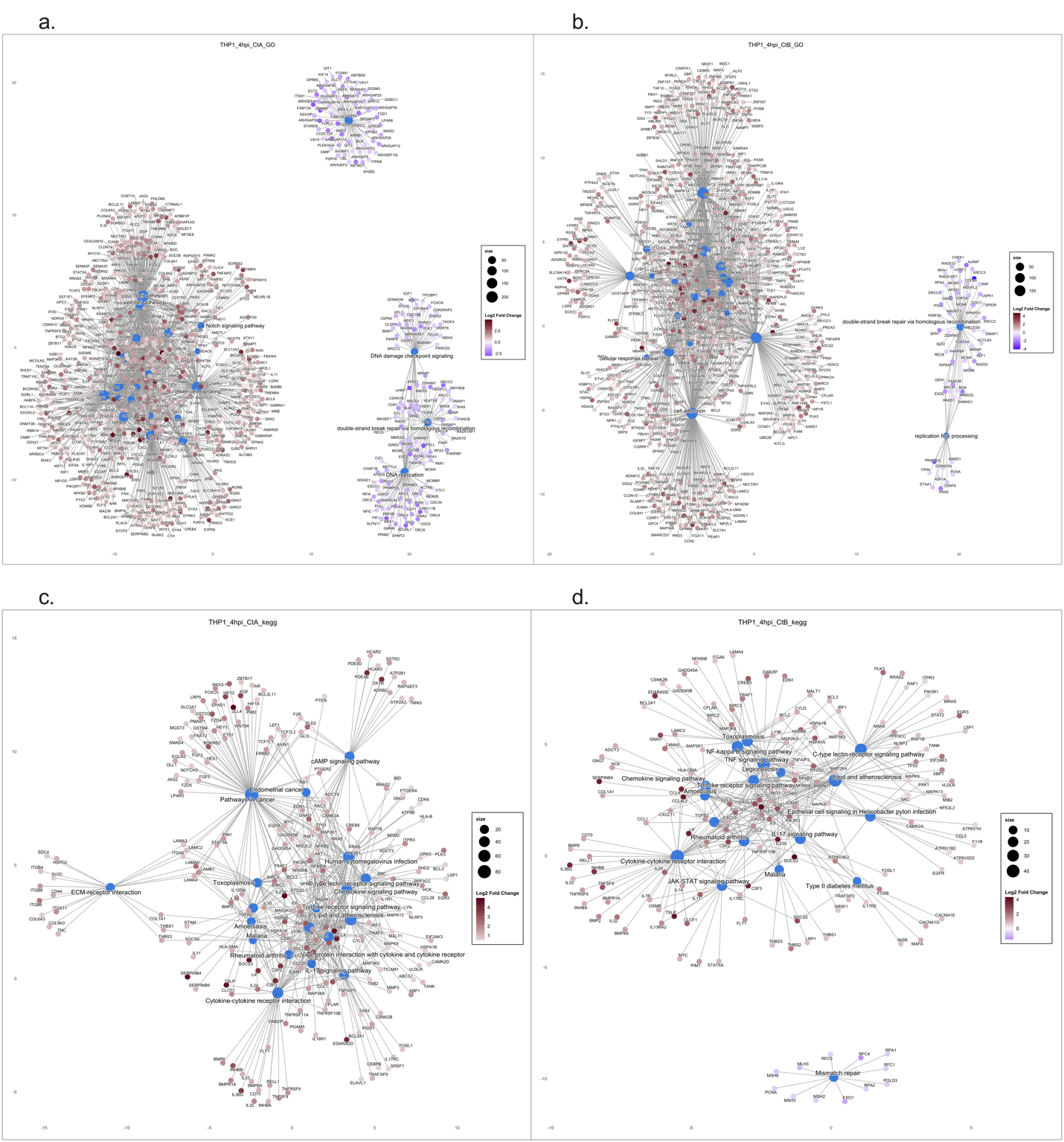
**

**Fig. S8.**

**
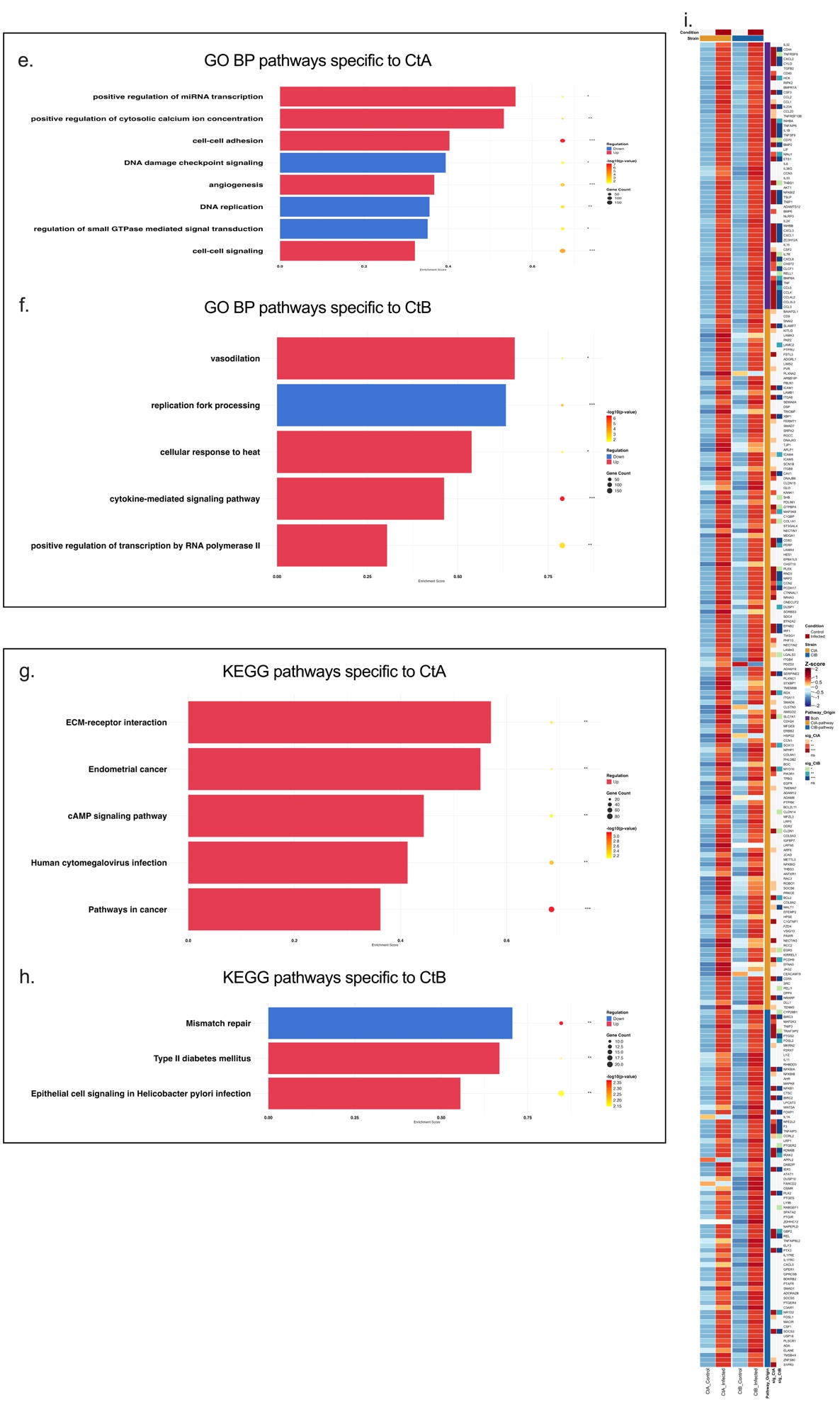
**

**Fig. S8.** Pathway enrichment analysis and differential gene expression profiles in THP-1 cells infected with Ct strains A/2497 and B/Tunis864 at 4 hpi. (**a**-**d**) Pathway-gene interaction networks for significantly enriched GO BP (**a**, **b**) and KEGG (**c**, **d**) pathways following CtA and CtB infection. Pathway nodes are sized by significance; gene nodes are coloured by log2FC (blue: downregulated; red: upregulated). (**e**-**h**) Strain-specific pathway enrichment profiles for GO BP (**e**, **f**) and KEGG (**g**, **h**). Bars show enrichment magnitude/direction; overlaid circles represent gene count (size) and significance (colour intensity, -log10 adjusted *P*-value). (**i**) Heatmap of differentially expressed pathway-associated genes (adjusted P < 0.05). Columns show mean Z-scored expression for controls and infected samples (CtA: A/2497; CtB: B/Tunis864). Row annotations indicate pathway origin (red: CtA-pathway; blue: CtB-pathway; orange: both) and strain-specific significance levels. Column annotations denote condition (grey: control; orange: infected) and strain (orange: CtA; blue: CtB). Colour scale: blue (low) to red (high) expression. Significance: *** *P* < 0.001, ** *P* < 0.01, * *P* < 0.05.

**
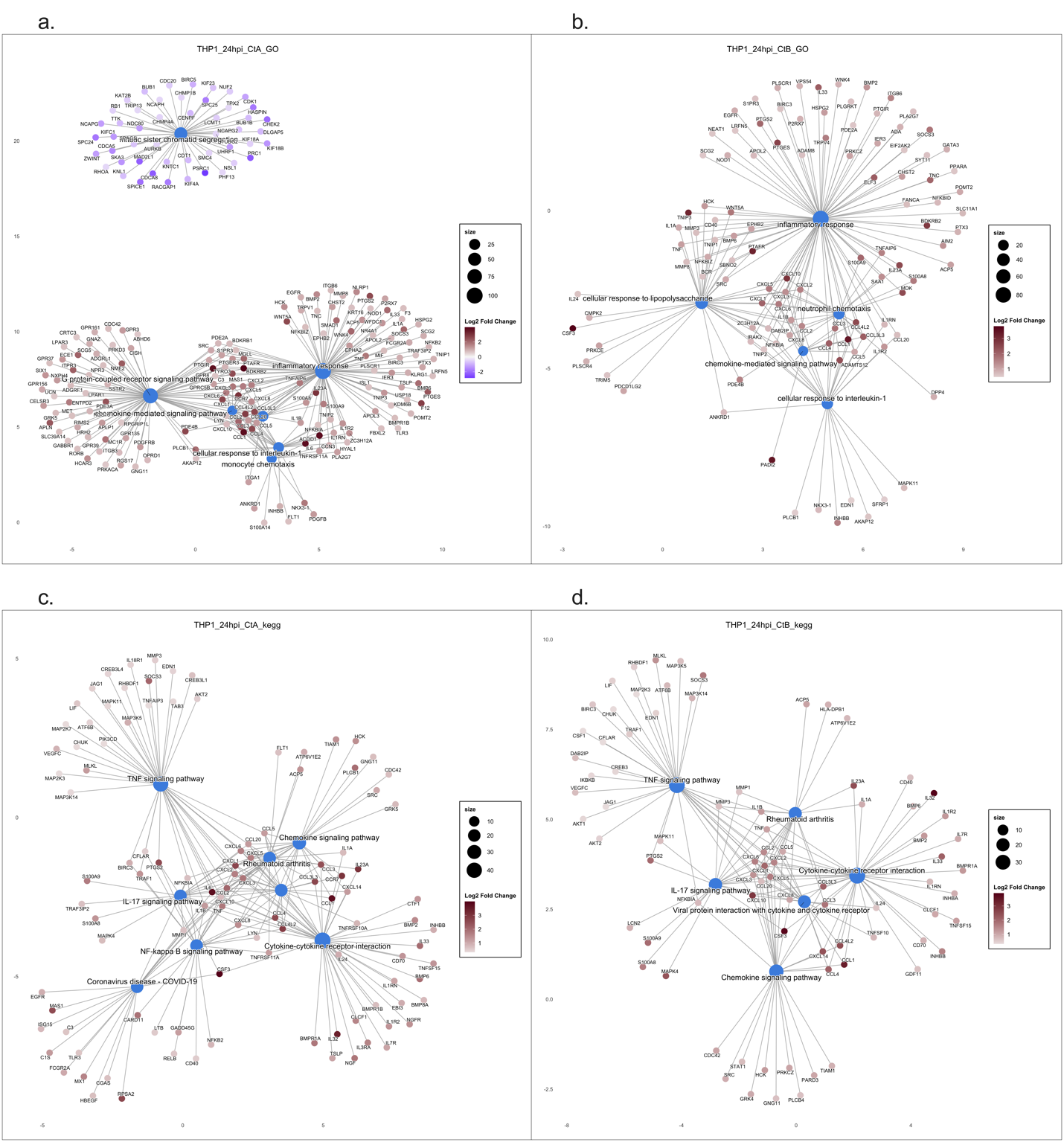
**

**Fig. S9.**


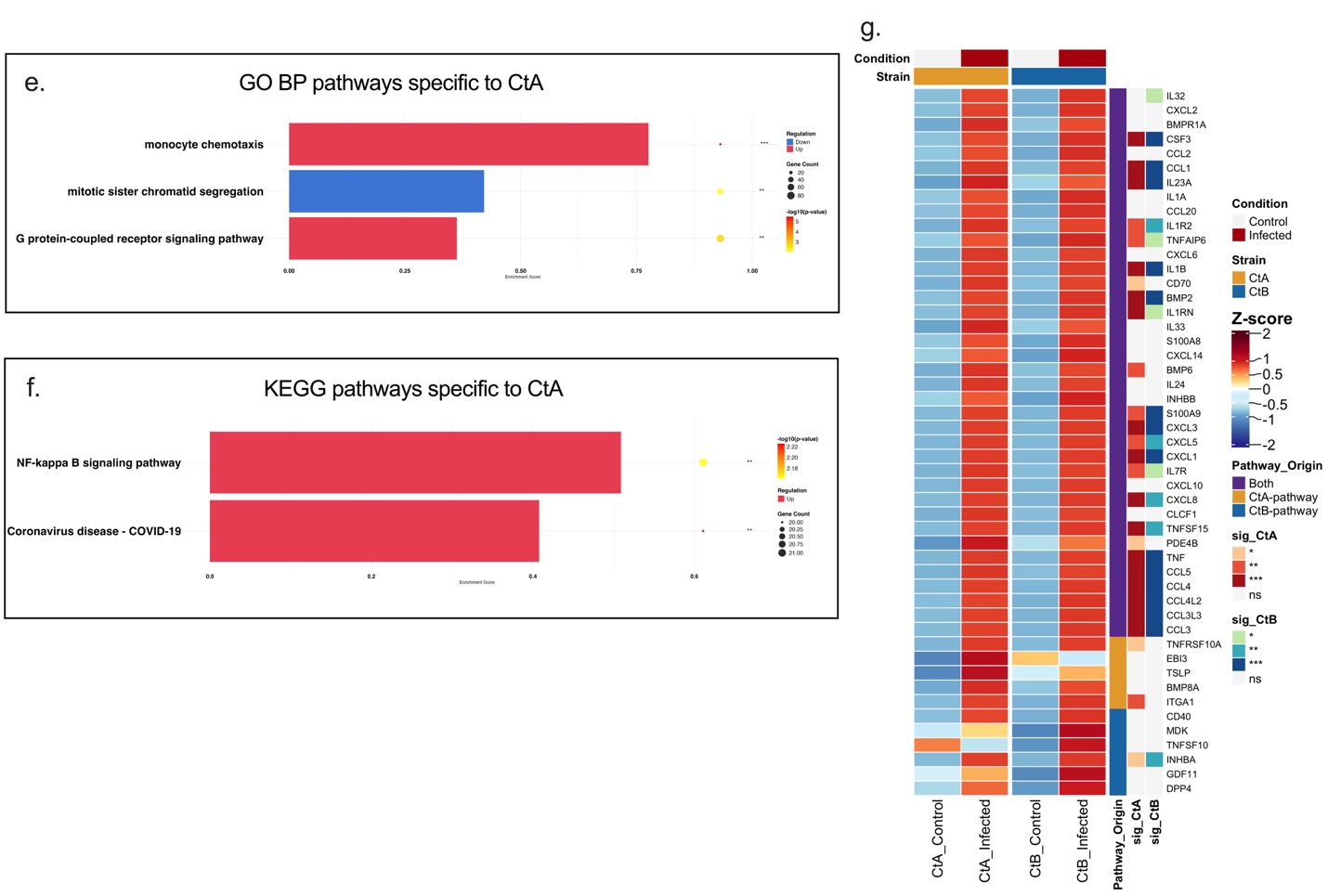
**Fig. S9.** Pathway enrichment analysis and differential gene expression profiles in THP-1 cells infected with Ct strains A/2497 and B/Tunis864 at 24 hpi. (**a**-**d**) Pathway-gene interaction networks for significantly enriched GO BP (**a**, **b**) and KEGG (**c**, **d**) pathways following CtA and CtB infection. Pathway nodes are sized by significance; gene nodes are coloured by log2FC (blue: downregulated; red: upregulated). (**e**-**f**) Strain-specific pathway enrichment profiles for GO BP (**e**) and KEGG (**f**). Bars show enrichment magnitude/direction; overlaid circles represent gene count (size) and significance (colour intensity, -log10 adjusted *P*-value). (**g**) Heatmap of differentially expressed pathway-associated genes (adjusted P < 0.05). Columns show mean Z-scored expression for controls and infected samples (CtA: A/2497; CtB: B/Tunis864). Row annotations indicate pathway origin (red: CtA-pathway; blue: CtB-pathway; orange: both) and strain-specific significance levels. Column annotations denote condition (grey: control; orange: infected) and strain (orange: CtA; blue: CtB). Colour scale: blue (low) to red (high) expression. Significance: *** *P* < 0.001, ** *P* < 0.01, * *P* < 0.05.
